# Strategic Secondary Ligand Selection for Enhanced Pore-Type Construction and Water Purification Capacity in Zeolitic Imidazolate Frameworks

**DOI:** 10.1021/acsami.4c21221

**Published:** 2025-03-30

**Authors:** Zheao Huang, Shaghayegh Naghdi, Adrian Ertl, Sabine Schwarz, Dominik Eder

**Affiliations:** 1 Institute of Materials Chemistry, Technische Universität Wien, Vienna 1060, Austria; 2 Service Center for Electron Microscopy (USTEM), Technische Universität Wien, Vienna 1040, Austria

**Keywords:** zeolitic imidazolate frameworks, selective ligand removal, mesopore, secondary ligand, water purification

## Abstract

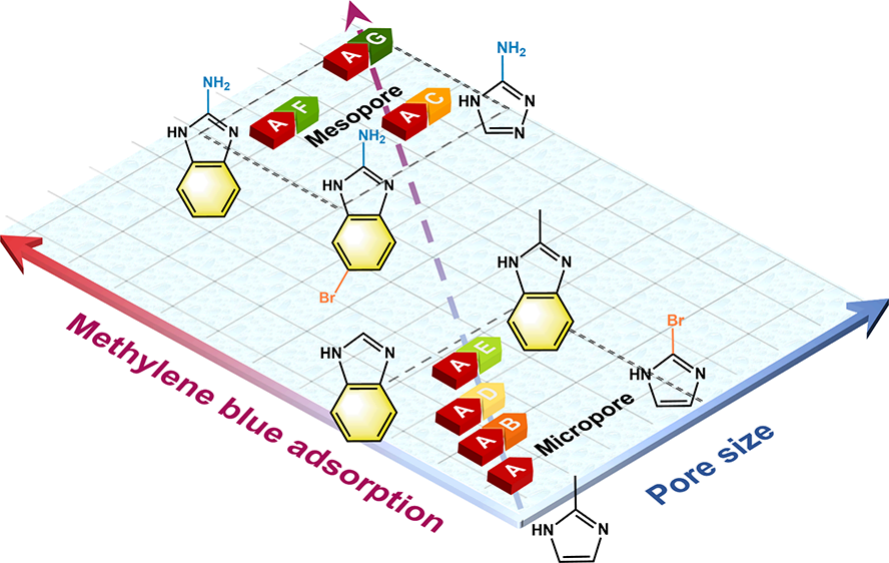

Selective ligand removal (SeLiRe) is a powerful strategy for constructing novel pore-type and ligand-defective structures in metal–organic frameworks (MOFs), but few studies have focused on the effect of secondary ligands with different functional groups on this process. We synthesized versions of zeolitic imidazolate framework-8 with six different secondary ligands and comprehensively investigated their pore-type structures after SeLiRe treatment. Their pore volume, size, and distribution are closely related to the respective organic functional groups on the secondary ligands. NH_2_-functionalized ligands tend to form larger domains and have weaker Zn–N_β_ covalent bonds, which facilitate the removal process and the construction of larger cavities. Among the six secondary ligands, 5-bromo-1*H*-benzo[*d*]imidazol-2-amine exhibits the composite pore-type structure with hierarchical micro- and mesopores, achieving the highest methylene blue adsorption capacity of 28.1 mg g^–1^. Compared to traditional sodalite-type ZIFs, this results in a 53-fold increase in water pollutant adsorption. This work highlights the crucial role of the secondary ligand in the SeLiRe strategy and provides valuable insights for designing other hierarchical porous hybrid structures.

## Introduction

Metal–organic frameworks (MOFs) are crystalline materials formed through the self-assembly of metal nodes (or clusters) with organic ligands, typically resulting in a porous periodic porous network. Their synthesis and applications have been extensively studied in materials science.^1,2^ Zeolitic imidazolate framework-8 (ZIF-8), a distinct subclass of MOFs, is composed of divalent Zn^2+^ ions coordinated with nitrogen atoms in imidazole ligands, adopting a zeolitic sodalite (SOD) topology with a characteristic microporous structure (pore size <2 nm).^3^^,^^4^ However, the absence of larger-scale cavities has been identified as a major kinetic limiting step, particularly for the adsorption and degradation of macromolecules/supramolecules, thereby constraining the multifunctionality and versatility of SOD-type ZIFs for broader applications.^5−8^ To address this challenge, the development of extended pore structures (such as mesopores, 2–50 nm) within parent ZIFs has become an urgent priority.

In our previous report, we synthesized mixed-ligand ZIF-8 (ML-ZIF) and used a highly selective ligand removal strategy (SeLiRe) to construct a series of uniform mesoporous ZIFs while maintaining the inherent crystal structure.^9^ However, in the report, we chose only 2-aminobenzimidazole as the secondary ligand, which did not extend the SeLiRe strategy to other common imidazolate ligands and other types of clusters. Similarly, most other reports on constructing mesopores or macropores through ligand removal have only attempted a single secondary ligand, without in-depth exploration of how different functional groups affect the selectivity, rate, and completeness of ligand removal.^10−14^ This oversight hinders the generalization of the SeLiRe strategy as a general approach within the MOF framework and makes it difficult to understand how various functional groups within the secondary ligand influence the coordination strength through electron donor/acceptor effects.

Herein, we selected six secondary ligands with diverse functional groups, such as aromatic carbon rings, −Br (bromine), −NH_2_ (amino group), and −CH_3_ (methyl group), to partially replace the primary ligand 2-methylimidazole (2-mIm). Using nuclear magnetic resonance (^1^H NMR) and infrared spectra, we successfully demonstrated the incorporation of secondary ligands while maintaining the sodalite-type ZIF structures. The thermal degradation window for secondary ligand removal was determined by thermogravimetric analysis. Subsequently, using the SeLiRe thermal treatment, we selectively removed the secondary ligands from the ML-ZIFs to construct the novel pore-type structures. Finally, the sizes, volumes, and distributions of the resulting hierarchical micro- and mesopores were evaluated using N_2_ physisorption measurements. Transmission electron microscopy, X-ray diffraction, and ^1^H NMR confirmed the successful thermal removal of the secondary ligands and that the samples retained good crystallinity. Based on the experimental conclusions, we inferred that the NH_2_-functionalized ligands can effectively expand the distribution of nanodomain defects, facilitating the formation of composite pore-type structures with hierarchical micro- and mesopores. Finally, we found that benefiting from the increased spatial availability and accessibility within the pore cavities of the ZIF framework, the best sample achieved a maximum adsorption capacity for methylene blue of 28.1 mg g^–1^, about 53 times that of traditional sodalite ZIFs.

## Results and Discussion

### Structural Characterization of Mixed-Ligand ZIFs

**Figure 1:**
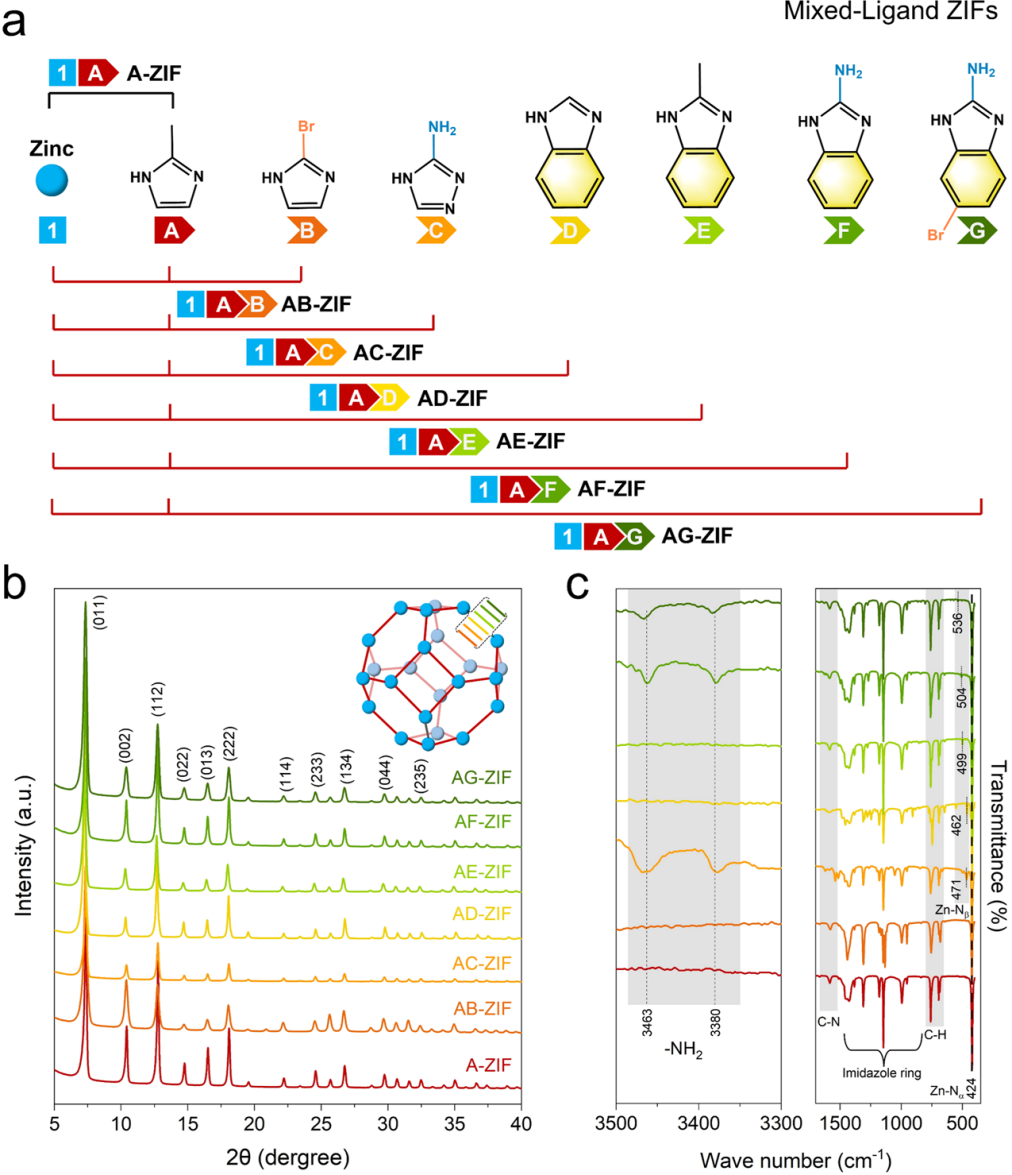
Structural characterization. (a) Schematic diagram of the various ligands A–G mixed in ML-ZIFs. (b) XRD and (c) ATR-IR of A-ZIF, AB-ZIF, AC-ZIF, AD-ZIF, AE-ZIF, AF-ZIF, and AG-ZIF.

The single-ligand ZIF-8 framework is formed by the tetrahedral coordination of zinc ions with the ligand 2-methylimidazole (2-mIm, referred to as A), resulting in the sodalite-type ZIF periodic structures. To ensure the effectiveness of the selective ligand removal strategy (SeLiRe), we introduced six imidazole-derived ligands (donated as B–G): 2-bromo-1*H*-imidazole (B), 3-amino-1,2,4-triazole (C), benzimidazole (D), 2-methylbenzimidazole (E), 2-aminobenzimidazole (F), and 5-bromo-1*H*-benzo[*d*]imidazol-2-amine (G). Each secondary ligand was mixed with the primary ligand A using solvent-assisted ligand exchange (SALE) to construct a series of mixed-ligand ZIFs (ML-ZIFs in Figure 1a). The X-ray powder diffraction (XRD) pattern of A-ZIF was in excellent agreement with the sodalite-type ZIF-8 reported in previous literature, characterized by multiple Bragg peaks with good crystallinity (Figure 1b and Figure S1).^15,16^ The Bragg peaks in the XRD patterns of the ML-ZIFs with various secondary ligands also correspond well to those of the A-ZIF, indicating that ligand substitution occurred solely within the framework and did not lead to additional impurity phases. Notably, the decreased Bragg peak intensity in some ML-ZIFs compared to A-ZIF reflects the competitive coordination between the secondary ligands and the primary ligand.

In the attenuated total reflection infrared (ATR-IR) spectra of ML-ZIFs, distinct from A-ZIF, the incorporation of secondary ligands results in the appearance of new bands in the range of 471 to 536 cm^–1^ (Figure 1c). These bands are located at 471 cm^–1^ (C), 462 cm^–1^ (D), 499 cm^–1^ (E), 504 cm^–1^ (F), and 536 cm^–1^ (G), closely resembling the Zn–N_α_ band (424 cm^–1^) of the primary ligand (A). Secondary ligand B did not show a new band from 472 to 505 cm^–1^ due to its structural similarity to the primary ligand A. Based on the previous reports on mixed-ligand ZIFs,^17,18^ these new bands are attributed to the binding of the nitrogen from the secondary ligand to the Zn ions, referred to herein as Zn–N_β_. Additionally, for secondary ligands C, F, and G, which contain −NH_2_ functional groups, additional ν(−NH_2_) modes can be observed (symmetric at 3380 cm^–1^ and asymmetric at 3463 cm^–1^) in the IR spectra. Variations in the imidazole ring stretching region (700 to 1500 cm^–1^) are also noted compared to A-ZIF due to the influence of the secondary ligands. In summary, the presence of additional ν(Zn–N_β_) and ν(−NH_2_) modes indicates the completion of the ligand substitution process, successfully integrating the secondary ligands into the ZIF framework.

In the ^1^H nuclear magnetic resonance (^1^H NMR) spectrum shown in Figure S2, the single-ligand A-ZIF exhibits the expected NMR characteristics with peaks 1 and 2 corresponding to the methyl and imidazole hydrogens on ligand A. For the other ML-ZIFs, new signals corresponding to the secondary ligands B–F (peaks 3–14 in Figures S3–S8) can be observed around peaks 1 and 2, and peaks 1 and 2 are also preserved. This indicates that both the primary ligand A and each of the secondary ligands (B–F) coexist in the ZIF framework.^19^ Quantitative analysis of these signals reveals the actual incorporation ratio of the secondary ligands: B is 19.5 mol %, C is 13.1 mol %, D is 27.3 mol %, E is 26.0 mol %, F is 13.9 mol %, and G is 14.7 mol %. Setting aside the direct competitive coordination effects of the ligands, the respective ratio of most ligands closely matches the nominal values (as detailed in Table S1), further ensuring the accuracy of the incorporation of secondary ligands into the ML-ZIFs.

Scanning electron microscopy (SEM) observations revealed that similar to the single-ligand A-ZIF, the single particles of all ML-ZIFs exhibited a dodecahedral morphology. Specifically, the average particle size distribution of samples AB-ZIF, AC-ZIF, AD-ZIF, and AE-ZIF is around 250 nm, while that of samples A-ZIF, AF-ZIF, and AG-ZIF is around 350 nm (Figures S9–S15). The variation in particle size can be attributed to the competitive coordination between the secondary ligand and the primary ligand, leading to the presence of structural distortion.^20,21^ This phenomenon is quite common in MOF ligand engineering, where the incorporation of a secondary ligand causes a certain degree of disorder compared to the prior state, ultimately affecting the morphology and the “optimal” particle size.^19^

### Selective Ligand Removal Strategy

**Figure 2:**
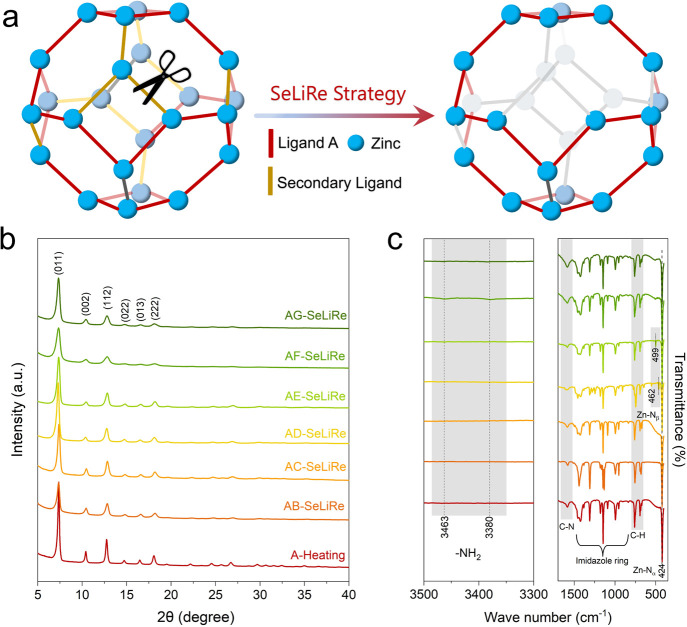
Structural characterization after the SeLiRe strategy. (a) Schematic diagram of the SeLiRe strategy. (b) XRD and (c) ATR-IR of A-Heating, AB-SeLiRe, AC-SeLiRe, AD-SeLiRe, AE-SeLiRe, AF-SeLiRe, and AG-SeLiRe.

The SeLiRe thermal treatment primarily involves the application of suitable temperatures to carefully cleave the metal–ligand coordination bonds, selectively removing the thermolabile secondary ligands from the ML-ZIFs (Figure 2a). Thermogravimetric analysis (TGA) was used to examine the thermal stability and temperature window of the imidazole-derived ligand (A–G) in the ML-ZIFs, providing essential information for the effective implementation of the SeLiRe strategy.^11^ The thermal stability of the corresponding ZIF frameworks decreases in the order A ≈ B > D ≈ E > F ≈ G ≈ C (Figure S16). This trend also reflects the ease of selectively removing imidazole-derived ligands with diverse functional groups through thermolysis; the thermolabile ligands, such as C, F, and G with −NH_2_ groups, are more easily removed. Guided by the weight loss information obtained from the TGA curves and our previous report,^9^ we can carefully select the suitable heating parameters, including temperature and time. This strategy aims to achieve thermal decomposition of the secondary ligands B–G while retaining the primary ligand A, thus avoiding the complete collapse of the inherent ZIF framework.

After the thermal treatment of the as-prepared ZIFs using the SeLiRe strategy, further analyses were conducted using XRD, SEM, and ATR-IR measurements. The reference sample, obtained using the same thermolytic conditions on A-ZIF, is named A-Heating. As shown in Figure 2b, all samples retained good crystallinity, with no disappearance of the Bragg peaks, indicating that the inherent ZIF framework was not damaged by the temperature. Meanwhile, the morphology in the SEM images mostly retained the typical dodecahedral structure of sodalite-type ZIF without causing any surface collapse or shrinkage (Figure S17). The retention of the Zn–N_α_ bonds and the stretching vibrations of the primary ligand A in the ATR-IR spectra further supports this, demonstrating that the SeLiRe thermal treatment does not seriously affect the crystal structure of the ZIF frameworks constructed by ligand A (Figure 2c). In contrast, the additional Zn–N_β_ and −NH_2_ stretching peaks caused by the secondary ligands C, F, and G were almost unidentifiable after the SeLiRe thermal treatment. These observations indicate that by precisely controlling the heating parameters, selective removal of the secondary ligands can be achieved through the cleavage of Zn–N_β_ bonds. It is noteworthy that the Zn–N_β_ bond in AD-SeLiRe and AE-SeLiRe did not show any weakening, indicating that ligands D and E were likely not successfully selectively removed. Consistent with the observations in the ^1^H NMR spectra, the signals for the secondary ligands C, F, and G nearly disappeared (0.0–5.5 mol %) after SeLiRe thermal treatment, while the signals for secondary ligands B, D, and E were mostly retained (Figures S2–S8 and Table S1).

Hence, the collective findings from TGA, XRD, ATR-IR, ^1^H NMR, and XRD analyses validate that SeLiRe thermal treatment of the mixed-ligands AC-ZIF, AF-ZIF, and AG-ZIF can achieve precise removal of the secondary ligands C, F, and G through the cleavage of Zn–N_β_ bonds while preserving the sodalite topology framework maintained by the primary ligand A. This strategy, however, is difficult to implement successfully for the secondary ligands B, D, and E in AB-ZIF, AD-ZIF, and AE-ZIF. The main reasons for this result are related to the differences in organic functional groups carried by the secondary ligands, which will be discussed in detail in the mechanism section below.

### Composite Pore-Type Construction in SeLiRe-ZIFs

**Figure 3:**
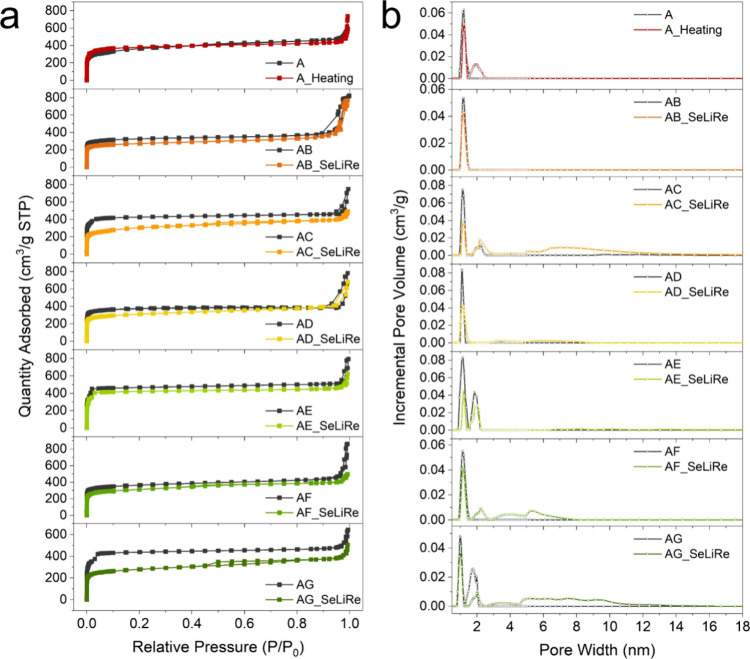
Pore-type structure characterization. (a) N_2_ physisorption isotherms and (b) porosity distributions of A-ZIF, A-Heating, AB-ZIF, AB-SeLiRe, AC-ZIF, AC-SeLiRe, AD-ZIF, AD-SeLiRe, AE-ZIF, AE-SeLiRe, AF-ZIF, AF-SeLiRe, AG-ZIF, and AG-SeLiRe.

The porosity parameters and Brunauer–Emmett–Teller (BET) specific surface area of the ML-ZIFs and their corresponding SeLiRe-ZIFs were evaluated through N_2_ physisorption at 77 K. Before the SeLiRe heat treatment, A-ZIF and all ML-ZIFs exhibit type-I isotherms, characteristic of typical microporous materials (Figure 3a). The distinct peak centered at 1.2 nm represents the intrinsic micropores (Figure 3b). Notably, 8 out of the 14 samples investigated have two low-pressure steps in their isotherms, with corresponding bimodal micropores in the pore size distribution at 0.5 to 2.2 nm (Figure S18). This does not indicate the formation of a new phase induced by a mixed-ligand strategy or thermal treatment. Instead, the additional steps can be attributed to the nitrogen adsorption characteristics during the measurement. Several factors may account for this, such as its quadrupole moment allowing interactions with polar sites within the ZIF framework, leading to an extra adsorption step at low pressure.^22^ Furthermore, the kinetic restrictions on nitrogen diffusion within microporous ZIFs may also contribute, causing adsorption to occur across different pressure ranges rather than in a single continuous process.

Due to the differences in nucleation competition between two ligands caused by different organic functional groups, the BET surface area of ML-ZIFs fluctuates slightly compared to the single-ligand A-ZIF, ranging from 1022.3 to 1309.9 m^2^ g^–1^ (Table S2). After the SeLiRe heat treatment, as expected, the inherent micropores of the SeLiRe-ZIFs series were partially retained. However, due to the detachment of the secondary ligand, both their micropore volume and BET surface area decreased slightly, while the final surface area remained at 807.7 to 1180.3 m^2^ g^–1^. The main difference from the ML-ZIFs before heat treatment is that AC-SeLiRe, AF-SeLiRe, and AG-SeLiRe exhibit a distinctly new pore-type structure and hysteresis loops, which are closely related to the secondary ligand and its corresponding functional groups (Figure S19). Specifically, AG-SeLiRe contains both aromatic carbon ring and −Br/−NH_2_ groups, resulting in a larger and more pronounced hysteresis loop compared to AC-SeLiRe and AF-SeLiRe. This can be attributed to the presence of multiple functional groups that influence the rigidity of the ZIF framework, leading to different defect formation pathways, thereby altering the SeLiRe efficiency and hysteresis behavior.^12,14^ Additionally, AG-SeLiRe is highly likely to contain more ink-bottle-shaped mesopores, which enhance capillary condensation and lead to a stronger hysteresis effect.^23^

Among the samples, AC-SeLiRe has the largest mesopores among the SeLiRe-ZIFs, with a pore diameter ranging from 2 to 17.8 nm and a volume of 0.231 cm^3^ g^–1^. The mesopores in AD-SeLiRe are either negligible or minimal with a slightly lower mesopore volume of 0.093 cm^3^ g^–1^. AF-SeLiRe and AG-SeLiRe exhibit a typical mesopore structure ranging from 2 to 8.2 and 2 to 16.7 nm, respectively, with similar mesopore volumes of 0.198 and 0.188 cm^3^ g^–1^. In the cases where significant mesopores appear, AC-SeLiRe, AF-SeLiRe, and AG-SeLiRe retain their inherent micropores, finally displaying the composite pore-type structure with hierarchical micro- and mesopores.

As a reference, the A-Heating sample forms only the micropore distribution without significant mesopores. This further confirms that the incorporation of the secondary ligand is crucial for mesopore formation, which cannot be achieved with the single-ligand A-ZIF. Based on previous experimental analysis, the SeLiRe thermal treatment was unable to remove ligands B and E effectively, which is further evidenced by the nearly unchanged NLDFT porosity distribution before and after heat treatment.

**Figure 4:**
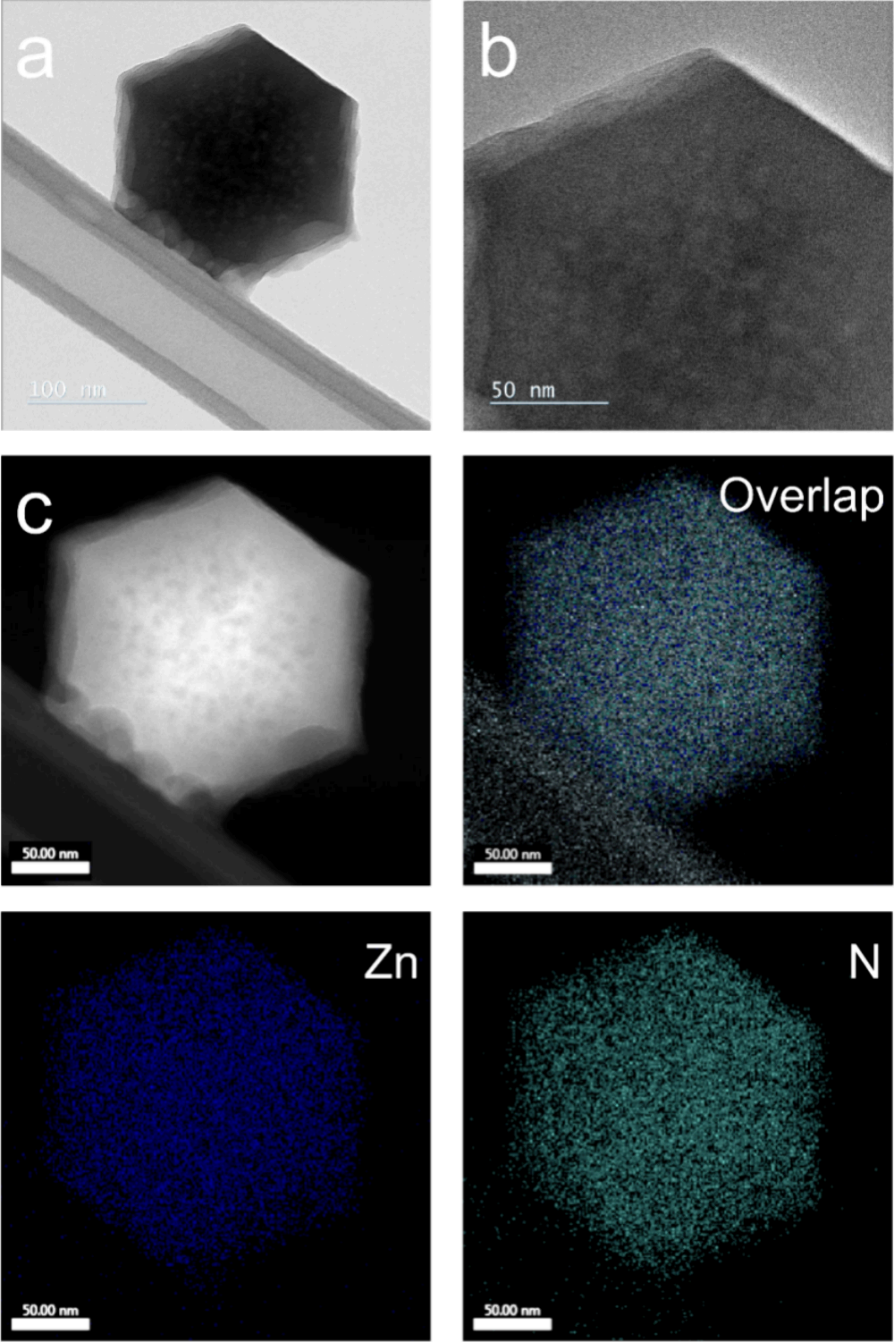
Morphology and element distribution of AG-SeLiRe. (a) TEM image of the single-particle AG-SeLiRe. (b) Enlarged TEM image of the AG-SeLiRe edge. (c) Elemental mapping of the single-particle AG-SeLiRe; EDS quantitative elemental analysis in Figure S20b.

Transmission electron microscopy (TEM) images of the AG-SeLiRe after heat treatment reveal a certain number of bright spots uniformly arranged in the particle centers, forming a sponge-like porous configuration (Figure 4a,b). Similarly, the clear vacancies in the element distribution can be seen in the energy-dispersive spectroscopy (EDS, see Figure 4c). This is distinctly different from the traditional sodalite-type ZIF and the TEM image of AG-ZIF before heat treatment (Figure S20a). Similar vacancies are also observed in AC-SeLiRe and AF-SeLiRe (Figures S21 and S22), further verifying that mesoscale voids can be uniformly constructed within the ZIF framework through the SeLiRe strategy. Note that it is difficult to accurately determine the pore size by TEM images due to the electron beam sensitivity of the MOF structures.^24^ However, these bright spots and elemental vacancies undoubtedly support the mesopore formation observed in the N_2_ physisorption analysis.

In conclusion, it can be inferred that the emergence of a composite pore-type structure with hierarchical micro- and mesopores, along with the volume, size, and distribution of SeLiRe-ZIFs under the same heating conditions, is greatly influenced by the secondary ligand incorporated during the initial synthesis process.

### Various Functional Groups of Secondary Ligands in Relation to the Domain Distribution

Validating the influence of various functional groups on the distribution and arrangement of secondary ligands is crucial for controlling the construction of novel pore-type structures in the ZIF framework. Previous studies have shown that the mixed-ligand ratio and the heating parameters can regulate the mesopores in microporous MOFs to a certain extent.^9,25^ Here, we primarily focus on the secondary ligands and their functional groups, striving to avoid any conditions that might affect the SeLiRe strategy and ensuring a comparison of SeLiRe-ZIFs under the same environment.

**Figure 5:**
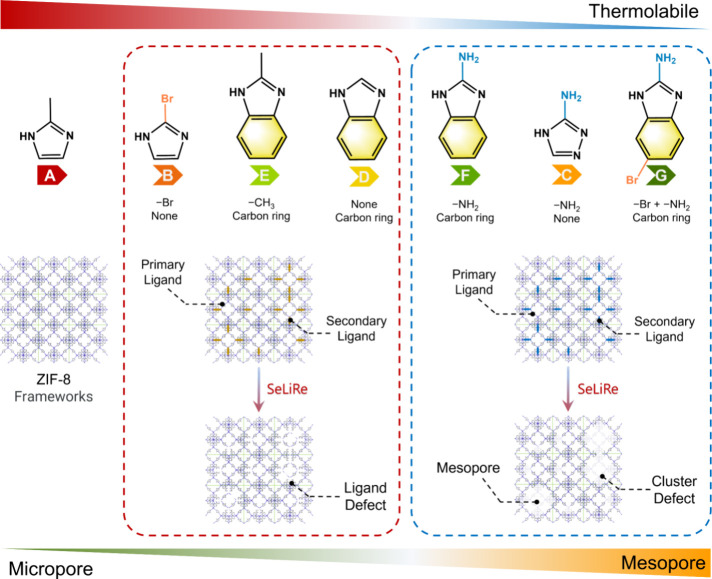
Mechanism of the pore-type structure. Schematic diagram for the secondary ligands in relation to the domain distribution.

According to the pore distribution data from N_2_ physisorption, it is evident that AC-SeLiRe, AF-SeLiRe, and AG-SeLiRe have all constructed the composite pore-type structures with hierarchical micro- and mesopores in the SOD topological ZIF framework. In contrast, this pore-type structure in other samples, such as A-Heating, AB-SeLiRe, AD-SeLiRe, and AE-SeLiRe, was unsatisfactory, with only inherent micropores observed, indicating the incomplete SeLiRe process. We deduce that the organic functional groups carried by each secondary ligand are the key factors causing these differences in pore-type structures, such as the mesopore size, volume, and inherent micropores. Specifically, secondary ligands C, F, and G with −NH_2_ groups can stably form significantly mesoporous structures after the removal of the ligand (Figure 5). This is difficult to achieve in secondary ligands B, E, and D, which contain only −Br, −CH_3_, and aromatic carbon rings. There are two possible reasons for this result: (1) secondary ligands with −NH_2_ groups are more likely to aggregate around ZnN_4_ clusters, creating larger domains that can be more easily removed to construct larger pore structures,^26,27^ and (2) the thermolabile nature of secondary ligands with −NH_2_ groups provides a larger thermal decomposition window. Notably, the simultaneous presence of multiple functional groups can further influence the rigidity of the ZIF framework, thereby affecting SeLiRe efficiency, ultimately resulting in larger and more distinct mesopores and hysteresis loops,^12,14^ as seen in AG-SeLiRe and its corresponding secondary ligand G (both aromatic carbon ring and −Br/−NH_2_ groups).

The SeLiRe heat treatment primarily achieves ligand removal through the cleavage of Zn–N_β_ bonds formed between the Zn metal and the nitrogen of the secondary ligand. Generally, NH_2_-functionalized ligands tend to weaken the Zn–N_β_ coordination bond, making them easier to remove under mild thermal treatment conditions.^9^ This is due to the electron-donating effect of the −NH_2_ group, which makes the imidazole ligand “harder”. Since ZIFs typically follow the HSAB theory (Hard and Soft Acid and Base Theory), assembling from soft divalent metal ions (Zn^2+^) and soft azolate ligands (imidazolates), a “harder” imidazole ligand reduces its binding affinity to Zn^2+^ ions.^28,29^ This weakens the corresponding Zn–N_β_ covalent bonds, making them more susceptible to thermal cleavage, as observed for AC-ZIF, AF-ZIF, and AG-ZIF. On the other hand, the introduction of the −NH_2_ group increases the electron density of the imidazole ring increases, while the electron cloud of the coordinating N atom with Zn^2+^ becomes more polarized. This may further weaken the bonding between the metal and the ligand, especially at high temperatures, where increased electron polarization could lead to structural loosening.

In contrast, secondary ligands B, E, and D, which contain only −Br, −CH_3_, and aromatic carbon rings, have none of the above features. Their thermal stability is similar to or close to that of the primary ligand A, leading to only ligand defects after thermal treatment without fully forming cluster defects from large domains, which completely hinders the construction of a composite pore-type structure by the SeLiRe thermal treatment. Of course, the formation of larger domains and even mesopores can be induced by stronger heating parameters or higher mixed-ligands ratios, but this would further damage the inherent crystallinity and microporous structure of the ZIF framework.

Therefore, in conclusion, when choosing to perform SeLiRe thermal treatment on ML-ZIFs to achieve the composite pore-type structures, we should prefer thermolabile imidazole ligands with −NH_2_ groups, which enable the construction of hierarchical micro- and mesoporous ZIFs with minimal loss under the lowest thermal treatment conditions.

### Dye Adsorption in Water Purification

To evaluate the beneficial potential of novel pore-type structures in SeLiRe-ZIFs for applications, we conducted water purification tests to adsorb the pollutant methylene blue (MB) from an aqueous solution. The adsorption kinetics and isotherm data best fit the pseudo-first-order (PFO) and Langmuir models, as detailed in Tables S3 and S4. This optimal fit typically indicates that the MB adsorption reactions on ZIF samples likely involve physisorption and monolayer molecular adsorption.^30,31^

**Figure 6:**
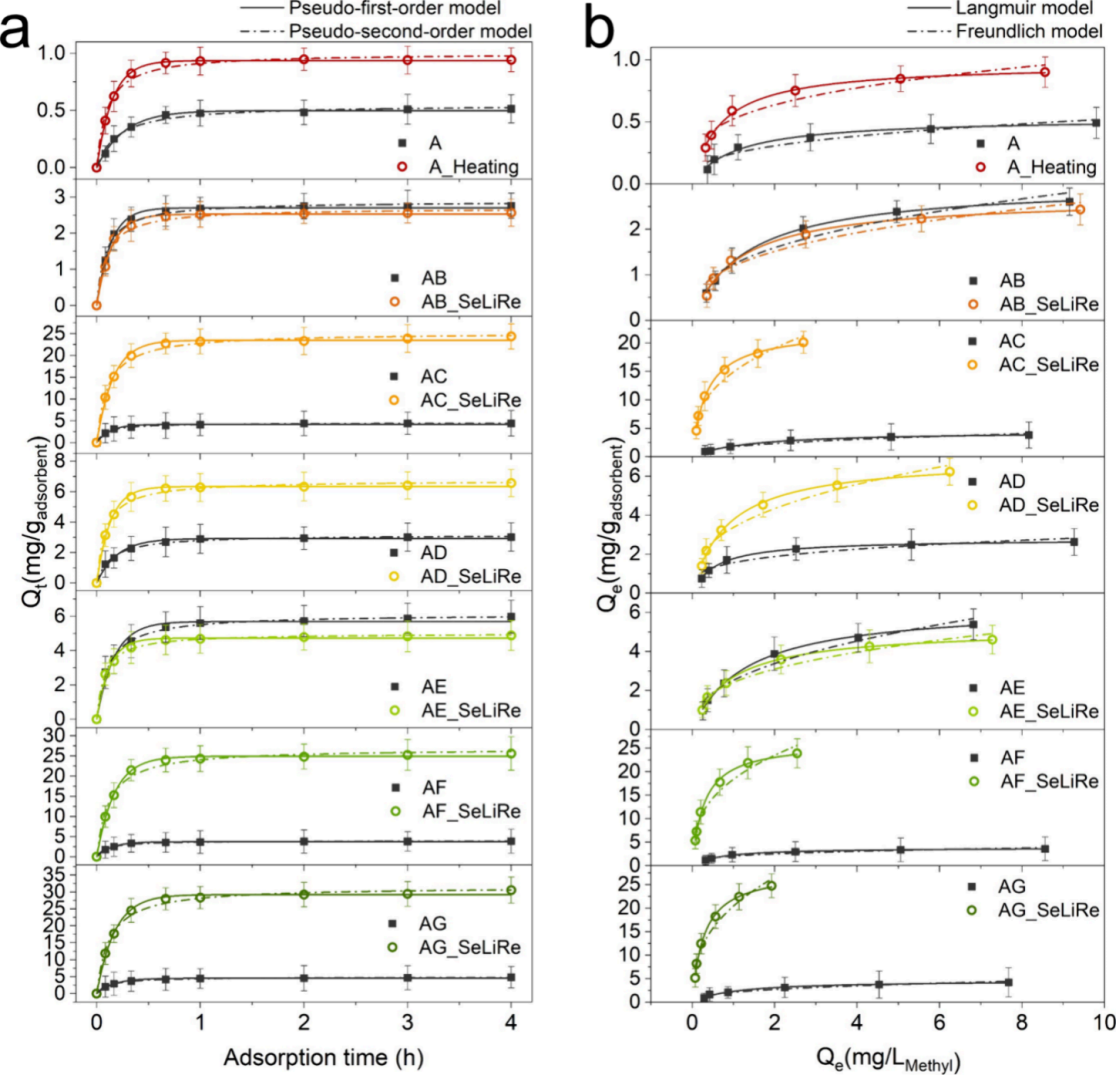
Methylene blue adsorption. (a) Kinetic curves and (b) isotherm curves of methylene blue for A-ZIF, A-Heating, AB-ZIF, AB-SeLiRe, AC-ZIF, AC-SeLiRe, AD-ZIF, AD-SeLiRe, AE-ZIF, AE-SeLiRe, AF-ZIF, AF-SeLiRe, AG-ZIF, and AG-SeLiRe.

In general, the narrow pore channels of traditional sodalite-type ZIFs hinder the diffusion of larger molecules like MB into their central cavities, significantly limiting the adsorption capacity of the samples.^9^ In contrast, the composite pore-type structures with hierarchical micro- and mesopores constructed through the SeLiRe strategy effectively eliminate this diffusion restriction, resulting in an increased adsorption performance. As expected, the adsorption kinetics and isotherm curves (Figure 6a,b) indicate that the A-ZIF and all ML-ZIFs have essentially minimal capacity for MB adsorption, with an adsorption capacity *Q*_m_ (maximum adsorption capacity) of only 0.531–6.35 mg g^–1^. However, upon removal of the secondary ligand and creation of the composite pore-type structures in the SeLiRe-ZIFs, their adsorption capacity is greatly enhanced. Specifically, the *Q*_m_ of AC-SeLiRe was 22.4 mg g^–1^, a 5-fold enhancement; that of AF-SeLiRe was 26.4 mg g^–1^, a 7-fold enhancement; and that of AG-SeLiRe was 28.1 mg g^–1^, a 6-fold enhancement in comparison to their respective ML-ZIFs, respectively. The adsorption capacity of these SeLiRe-ZIFs significantly surpasses that of conventional Cu-BDC^32^ and HKUST-1^33^ and is nearly comparable to the advanced Co-based MOF Co-UMO-1.^34^ This underscores the importance of using the SeLiRe strategy to expand the pore sizes of SOD-type ZIFs. For the other samples, such as A-Heating and AD-SeLiRe, only negligible increases are shown, while AB-SeLiRe and AE-SeLiRe do not exhibit noticeable changes. These adsorption performance results correlate with the pore-type structures obtained from N_2_ physisorption, indicating that the adsorption capacity significantly increases with the presence of additional mesopore distributions.

The ATR-IR spectra of AG-SeLiRe after MB saturated adsorption show neither the emergence of new bands nor notable shifts in the existing ones, further confirming that the primary mode of dye adsorption is physisorption (Figure S23). Additionally, the evaluation based on powder XRD data and six cycling tests effectively demonstrates that the AG-SeLiRe sample can serve as an efficient and durable adsorbent (Figure S24).

Based on the above analysis, the mechanism behind the enhanced adsorption can be determined. Since MB molecules are larger than the pore windows of pristine ZIF-8, their diffusion into the larger central cavities is significantly hindered. This strongly suggests that MB adsorption is kinetically restricted. Based on the isotherm fitting and the IR spectra after adsorption (Figure 6 and Figure S23), the adsorption process is primarily physisorption, further confirming that adsorption is kinetically driven. The MB adsorption capacity of mesoporous ZIF samples (AC-SeLiRe, AF-SeLiRe, and AG-SeLiRe in Figure 6) is about 5–7 times higher than that of nonmesoporous ZIF samples, with AG-SeLiRe exhibiting the highest adsorption performance, i.e, 53 times higher than that of A-ZIF. This enhancement indicates that the presence of larger pores or improved pore connectivity is key to effectively reducing diffusion limitations for MB molecules. Furthermore, the formation of mesopores also facilitates enhanced molecular transport, which can improve the reaction kinetics and size selectivity, making SeLiRe-ZIF highly promising for applications in gas separation, sensing, and membrane-based filtration.

In contrast, due to the dominance of physisorption and kinetic limitations, the role of open active sites or defects formed after ligand removal in dye adsorption is minimal. However, this does not mean that they are unimportant in other reactions, particularly in catalysis (photocatalysis and electrocatalysis). In our previous publication,^35^ the open metal sites have been demonstrated to significantly enhance the performance of the hydrogen evolution reaction (HER) in electrocatalysis, and we have conducted an in-depth study of their catalytic mechanisms.

## Conclusions

In summary, to investigate the influence of various ligands and their functional groups on the construction of composite pore-type structures via the selective ligand removal strategy (SeLiRe), we selected six secondary ligands, B–F, to be mixed with primary ligand A, synthesizing a series of mixed-ligand ZIF materials. Based on N_2_ physisorption data, after heat treatment with suitable parameters, the secondary ligands in some ML-ZIFs were thermally decomposed, forming additional mesoporous structures in the ZIF framework. We found that the emergence of the composite pore-type structure with hierarchical micro- and mesopores, along with their volume, size, and distribution, is greatly influenced by the secondary ligands and their different organic functional groups. Among them, the NH_2_-functionalized ligands (C, F, and G) achieved suitable mesopores ranging from 2 to 18 nm, while fully retaining the inherent crystallinity and micropore distribution of sodalite-type ZIF structures. Compared to other functional groups such as −Br, −CH_3_, and aromatic carbon rings, the −NH_2_ group strongly supports the idea that the additional mesopore formation through the SeLiRe strategy is mainly due to the formation of larger domains by ligand aggregation and the ease of cleavage of its weaker Zn–N_β_ bonds.

As expected, the adsorption capacity of SeLiRe-ZIFs with a composite pore-type structure for organic pollution in aqueous media significantly increased. Notably, the AG-SeLiRe sample exhibited the highest adsorption capacity for methylene blue, with a *Q*_m_ of 28.1 mg g^–1^, which is 53 times higher than that of A-ZIF. This enhancement is primarily attributed to the incorporation of the composite pore-type structure with hierarchical micro- and mesopores by secondary ligand removal, which provide larger pore activities and facilitate the rapid transport of dye molecules. Essentially, our work provides insights into the functionalization of secondary ligands for designing hierarchical micro- and mesoporous ZIFs with novel pore-type topologies.

## Experimental Section, Methods, and Materials

### Chemicals

The following materials were used: zinc nitrate hexahydrate (Zn(NO_3_)_2_·6H_2_O, 98%, ACROS), 2-methylimidazole (C_4_H_6_N_2_, 99%, Sigma-Aldrich), 2-bromo-1*H*-imidazole (C_3_H_3_BrN_2_, 95%, abcr GmbH), 3-amino-1,2,4-triazole (C_2_H_4_N_4_, 98%, TCL), benzimidazole (C_7_H_6_N_2_, 98%, Sigma-Aldrich), 2-methylbenzimidazole (C_8_H_8_N_2_, 98%, abcr GmbH), 2-aminobenzimidazole (C_7_H_7_N_3_, 97%, Sigma-Aldrich), 5-bromo-1*H*-benzo[*d*]imidazol-2-amine (C_7_H_6_BrN_3_, 95%, abcr GmbH), methanol (MeOH, 99.9%, HiPerSolv CHROMANORM, VWR), *d*_4_-acetic acid for ^1^H NMR (CD_3_CO_2_D, 99.4%, Thermo scientific), and methylene blue (MB, C_16_H_18_CIN_3_S·*x*H_2_O, high purity, biological stain, Alfa Aesar).

### Characterization

X-ray diffraction (XRD) was performed using a PANalytical X’Pert Pro multipurpose diffractometer (MPD) in Bragg–Brentano geometry equipped with a Cu anode operating at 45 kV and 40 mA. Attenuated total reflection-infrared spectroscopy (ATR-IR) was conducted with a PerkinElmer Spectrum Two FTIR spectrometer covering the infrared range of 400–4000 cm^–1^ and utilizing a LiTaO_3_ (lithium tantalate) MIR detector. Nitrogen physisorption analysis was carried out at 77 K on a Micromeritics 3Flex analyzer, with samples pretreated by vacuum outgassing at 150 °C for 3–12 h. Scanning electron microscopy (SEM) was performed using an FEI Quanta 250 (Schottky) FEG-SEM instrument with support from USTEM at TU Wien. Transmission electron microscopy (TEM) was conducted with a Tecnai F20 FEG-TEM, also facilitated by USTEM at TU Wien. Nuclear magnetic resonance spectroscopy (^1^H NMR) was recorded on a Bruker ADVANCE 250 (250.13 MHz) instrument featuring a 5 mm inverse-broad probe head and a *z*-gradient unit. Thermogravimetric analysis (TGA) was carried out on a PerkinElmer 8000 (Waltham, USA) using Al_2_O_3_ crucibles. Samples were heated at a rate of 10 °C/min under air, argon, or nitrogen flow and held at 600 °C for 1 h. For more details, please refer to the Supporting Information.

### A-ZIF (Single-Ligand ZIF)

A solution of 2 g of 2-methylimidazole in 10 mL of HPLC-grade methanol was prepared, while 0.87 g of Zn(NO_3_)_2_·6H_2_O was separately dissolved in an equal volume of the same solvent. The two solutions were combined in a microwave synthesis reactor (Monowave 300, Anton Paar) and heated at 150 °C for 2 h to promote A-ZIF crystal formation. After standing for 24 h, the resulting ZIF powder was collected by centrifugation, washed three times with methanol and deionized water, and subsequently vacuum-dried in an oven overnight, yielding the final A-ZIF powder.

### Mixed-Ligand ZIFs

The ML-ZIFs (AB-ZIF, AC-ZIF, AD-ZIF, AE-ZIF, AF-ZIF, and AG-ZIF) were synthesized using the same procedure, with only the secondary ligand being varied accordingly. A mixture of 0.6 g of the secondary ligand (**B**, 2-bromo-1*H*-imidazole; **C**, 3-amino-1,2,4-triazole; **D**, benzimidazole; **E**, 2-methylbenzimidazole; **F**, 2-aminobenzimidazole; and **G**, 5-bromo-1*H*-benzo[*d*]imidazol-2-amine) and 1.4 g of 2-methylimidazole (**A**, the primary ligand) was dissolved in 10 mL of HPLC-grade methanol and heated to 100 °C for 30 min in a microwave synthesis reactor (Monowave 300, Anton Paar) to ensure complete dissolution. Meanwhile, 0.87 g of Zn(NO_3_)_2_·6H_2_O was dissolved in 10 mL of HPLC-grade methanol, sonicated for 10 min, and stirred for 30 min. The two solutions were then combined and heated at 150 °C for 2 h in the microwave reactor to promote ZIF crystal formation. After standing for 24 h, the precipitate was collected by centrifugation, washed three times with methanol and deionized water, and vacuum-dried overnight to obtain the purified powder.

### A-Heating and SeLiRe-ZIFs

The synthesized ZIF powders were thermally decomposed at 500 °C for 2 h under an argon atmosphere. This process was conducted in a tube furnace (HTM Reetz LK-1100, Germany) with a controlled heating ramp of 10 °C/min and a constant argon flow rate. After the target temperature was reached, the furnace was allowed to cool naturally to room temperature. All samples underwent thermal treatment under the same conditions. Depending on the precursor used, the resulting products were designated as A-Heating, AB-SeLiRe, AC-SeLiRe, AD-SeLiRe, AE-SeLiRe, AF-SeLiRe, and AG-SeLiRe.

### Adsorption Experiment Preparation

Before the adsorption tests were conducted, all ZIF adsorbents were dried at 60 °C overnight. For each experiment, 20 mg of adsorbent was introduced into 50 mL of a methylene blue (MB) solution at a concentration of 10 mg L^–1^ prepared with deionized (DI) water. This mixture was stirred continuously. At set intervals, ranging from 5 min to 4 h, a 1 mL sample of the solution was withdrawn for centrifugation, and the absorbance of the supernatant was measured across the 400–800 nm spectrum. The adsorption capacity of the adsorbent was calculated as follows:
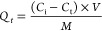

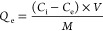


Here, *Q*_*t*_ (mg g^–1^) is the adsorption capacity for time *t*, where *C*_i_ and *C*_*t*_ denote the initial and specific time *t* concentrations (mg L^–1^) of MB, as determined via UV–visible spectroscopy of the absorbance. *Q*_e_ and *C*_e_ indicate the equilibrium adsorption capacity and concentration, respectively. *V* and *M* correspond to the volume of the MB solution (L) and the mass of the ZIF adsorbent (g).

### Adsorption Kinetics

The adsorption kinetics over time was fitted to pseudo-first-order (PFO) and pseudo-second-order (PSO) models,^30^ as described below:
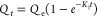

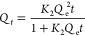


Here, *K*_1_ (min^–1^) and *K*_2_ (g mg^–1^ min^–1^) are rate constants associated with the PFO and PSO models, respectively.

### Adsorption Isotherms

For isothermal analysis, ZIF adsorbents were also dried at 60 °C overnight before each experiment. A 20 mg portion of adsorbent was added to an MB solution at varying concentrations (1–10 mg L^–1^). After 2 h of stirring, the adsorption was considered to reach saturation based on the adsorption kinetics. The equilibrium concentration *C*_e_ and adsorption capacity *Q*_e_ were derived from UV–vis absorbance data, and adsorption isotherms were generated. The data were fitted to Langmuir and Freundlich isotherm models,^36^ defined as follows:
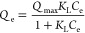

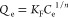


Here, *Q*_max_ (mg g^–1^) represents the maximum adsorption capacity. *K*_L_ (L mg^–1^) and *K*_F_ (mg g^–1^) (mg L^–1^)^−*n*^ are constants for the Langmuir and Freundlich models, representing the adsorption capacity and the adsorbate–adsorbent interaction strength. The parameter *n* describes adsorption intensity, reflecting the energy distribution and heterogeneity of adsorption sites.^37^ The Langmuir model represents the monolayer molecular adsorption on homogeneous surfaces, and the Freundlich model represents the multilayer adsorption on heterogeneous surfaces^31^

## Data Availability

All data supporting the findings of this study are available within the article and its Supporting Information file, as well as from the corresponding author upon reasonable request.

## Supporting Information Available

XRD patterns of 5–20° (Figure S1); ^1^H NMR of all samples (Figures S2–S8); SEM images of all samples (Figures S8–S15 and S17); TGA curves of ML-ZIFs (Figure S16); semilog plots and hysteresis loops of physisorption isotherms (Figures S18 and S19); TEM/EDS images of AG-ZIF, AC-SeLiRe, and AF-SeLiRe (Figures S20–S22); ATR-IR spectra of AG-SeLiRe before and after MB adsorption (Figure S23); XRD patterns of AG-SeLiRe before and after MB adsorption and adsorption–desorption cycles (Figure S24); ^1^H NMR quantitative analysis (Table S1); specific surface areas and porosity parameters of all samples (Table S2); kinetic parameters of all samples (Table S3); isotherm parameters of all samples (Table S4); and characterization details (PDF)
