# Supplementary material for: Strategic Secondary Ligand Selection for Enhanced Pore-Type Construction and Water Purification Capacity in Zeolitic Imidazolate Frameworks
Source: ACS Appl Mater Interfaces. 2025 Mar 30;17(14):21133–42. doi: 10.1021/acsami.4c21221 (PMC11986909; doi:10.1021/acsami.4c21221)
Supplement: Supplementary file 1 — am4c21221_si_001.pdf [file am4c21221_si_001.pdf]

# Supporting Information

# Strategic Secondary Ligand Selection for Enhanced Pore-Type Construction and Water Purification Capacity in Zeolitic Imidazolate Frameworks

*Zheao Huang [*,1], Shaghayegh Naghdi [1], Adrian Ertl [1], Sabine Schwarz [2], and Dominik Eder [*,1]*

[1] Institute of Materials Chemistry, Technische Universität Wien, 1060, Vienna, Austria

[2] Service Center for Electron Microscopy (USTEM), Technische Universität Wien, 1040, Vienna, Austria

* Corresponding authors: zheao.huang@tuwien.ac.at and dominik.eder@tuwien.ac.at

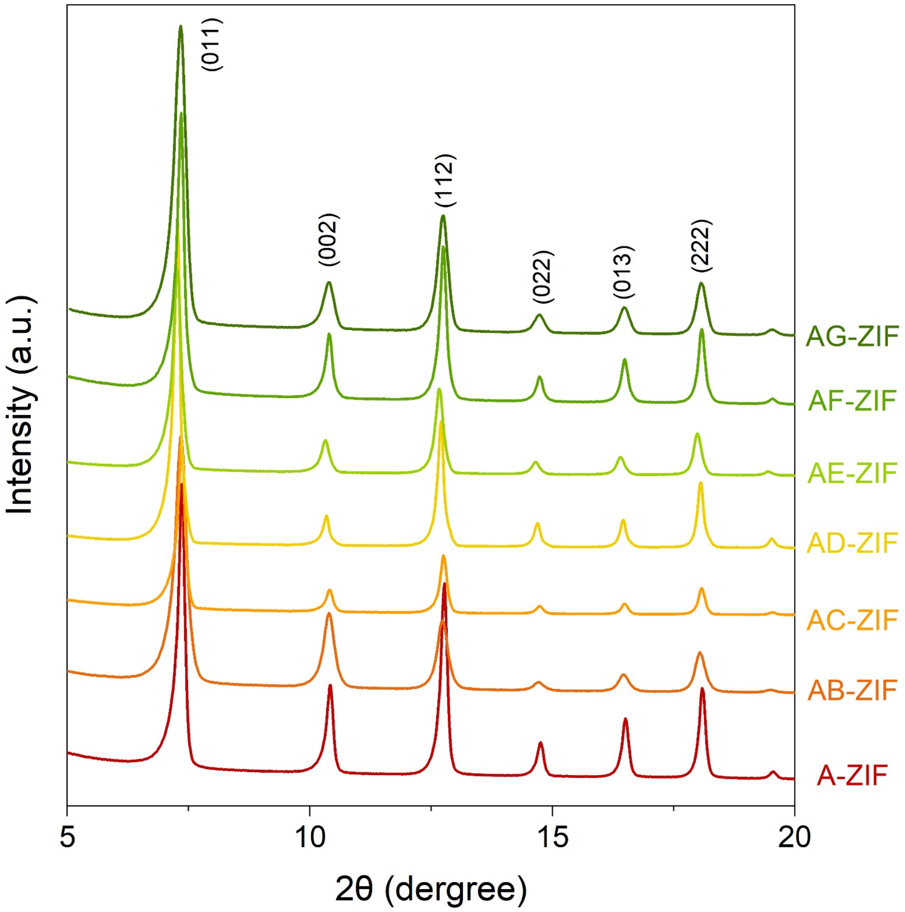


**Figure S1.** XRD patterns of A-ZIF, AB-ZIF, AC-ZIF, AD-ZIF, AE-ZIF, AF-ZIF and AG-ZIF in the lower x axis ranges (5-20 degree).

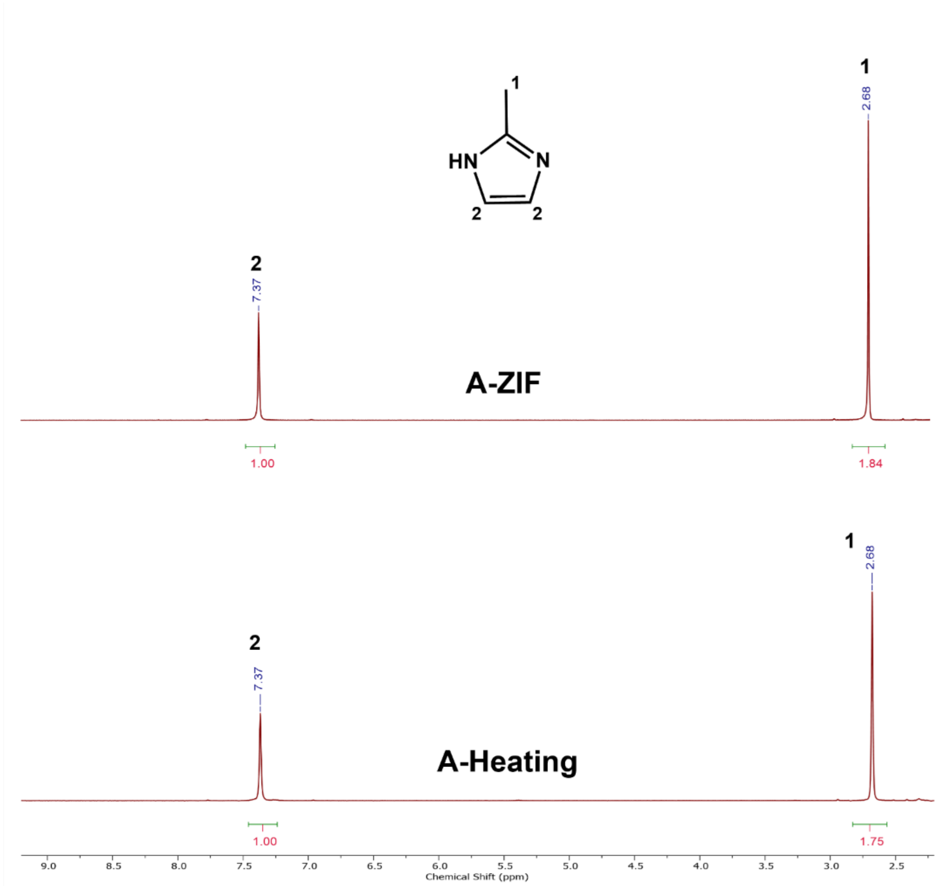


**Figure S2.** $^{1}$H NMR of A-ZIF and A-Heating.

A-ZIF: *$^{1}$H NMR (250 MHz, Acetic Acid-$d_4$) δ 7.37 (s, 1H), 2.68 (s, 2H).* A-Heating: *$^{1}$H NMR (250 MHz, Acetic Acid-$d_4$) δ 7.37 (s, 1H), 2.68 (s, 2H).*

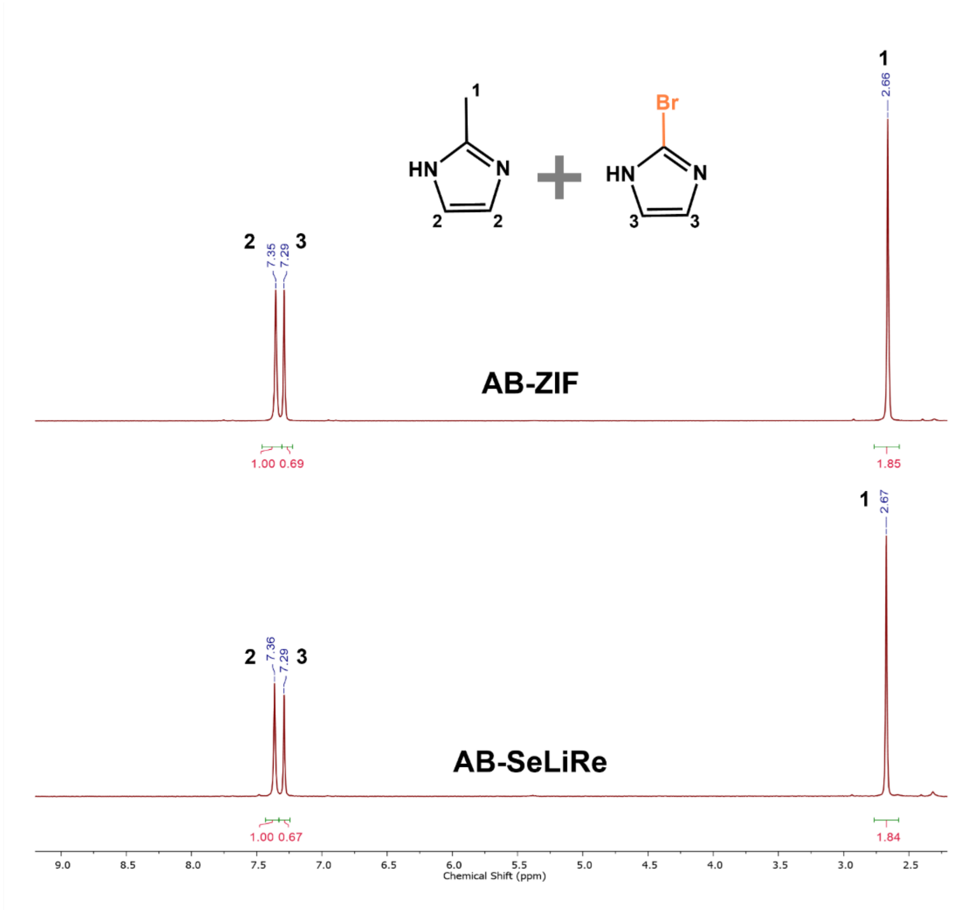


**Figure S3.** $^{1}$H NMR of AB-ZIF and AB-SeLiRe.

AB-ZIF: *$^{1}$H NMR (250 MHz, Acetic Acid-$d_4$) δ 7.35 (s, 1H), 7.29 (s, 1H), 2.66 (s, 2H).* AB-SeLiRe: *$^{1}$H NMR (250 MHz, Acetic Acid-$d_4$) δ 7.36 (s, 1H), 7.29 (s, 1H), 2.67 (s, 2H).*

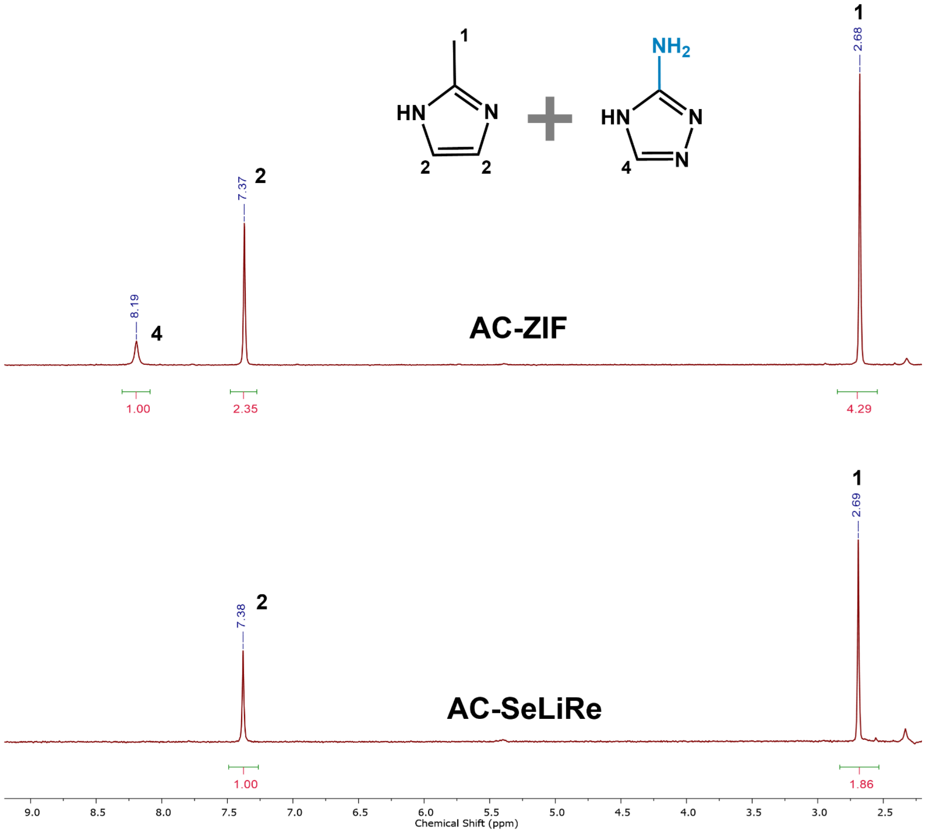


**Figure S4.** $^1$H NMR of AC-ZIF and AC-SeLiRe.

AC-ZIF: *$^1$H NMR (250 MHz, Acetic Acid-$d_4$) δ 7.42 (dd, J = 6.0, 3.2 Hz, 1H), 7.36 (s, 5H), 7.24 (dd, J = 6.0, 3.2 Hz, 1H), 2.67 (s, 9H)*. AC-SeLiRe: *$^1$H NMR (250 MHz, Acetic Acid-$d_4$) δ 7.44 (d, J = 14.2 Hz, 1H), 7.37 (s, 6H), 2.68 (s, 11H).*

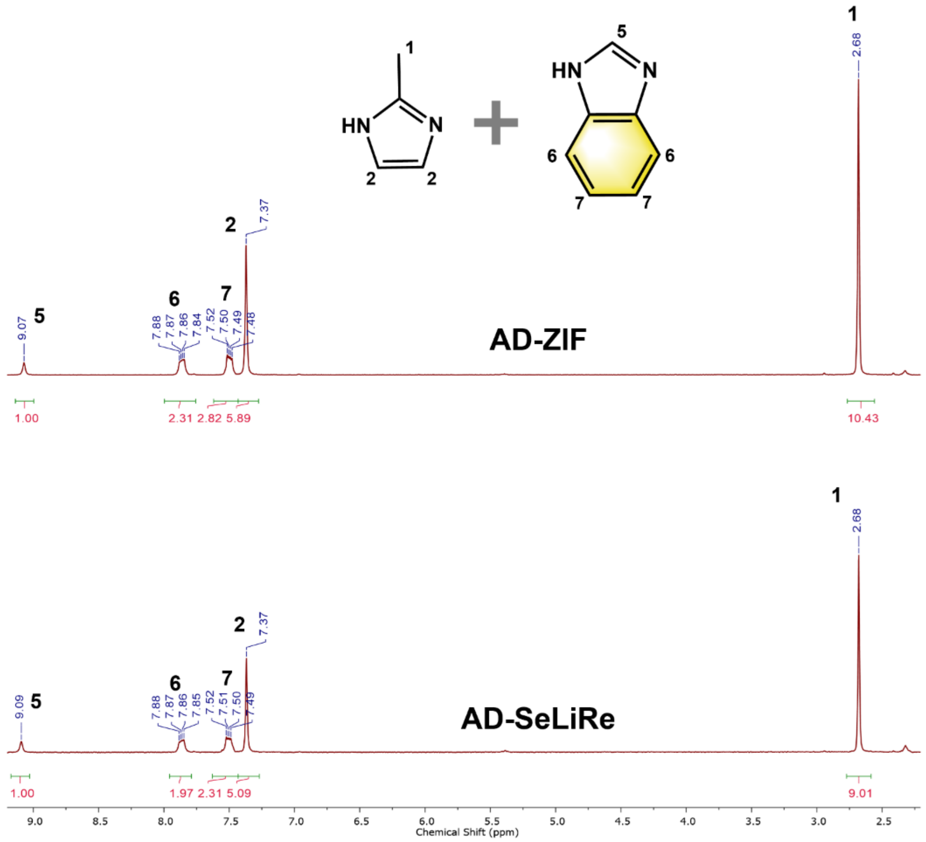


**Figure S5.** $^{1}$H NMR of AD-ZIF and AD-SeLiRe.

AD-ZIF: *$^{1}$H NMR (250 MHz, Acetic Acid-$d_4$) δ 8.19 (s, 1H), 7.37 (s, 2H), 2.68 (s, 4H).* AD-SeLiRe: *$^{1}$H NMR (250 MHz, Acetic Acid-$d_4$) 7.38 (s, 1H), 2.69 (s, 2H).*

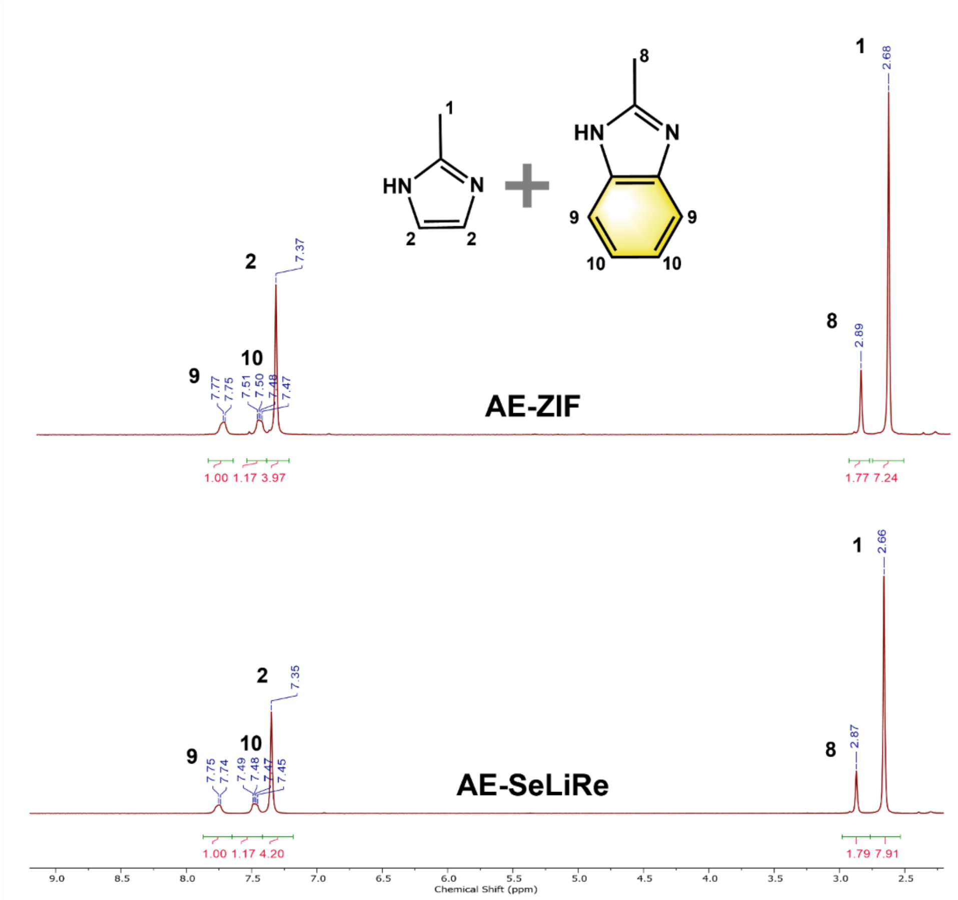


**Figure S6.** $^1$H NMR of AE-ZIF and AE-SeLiRe.

AE-ZIF: *$^1$H NMR (250 MHz, Acetic Acid-$d_4$) δ 9.07 (s, 1H), 7.86 (dd, J = 6.2, 3.2 Hz, 2H), 7.50 (dd, J = 6.3, 3.2 Hz, 3H), 7.37 (s, 6H), 2.68 (s, 10H).* AE-SeLiRe: *$^1$H NMR (250 MHz, Acetic Acid-$d_4$) δ 9.09 (s, 1H), 7.87 (dd, J = 6.1, 3.2 Hz, 2H), 7.50 (dd, J = 6.2, 3.2 Hz, 2H), 7.37 (s, 5H), 2.68 (s, 9H).*

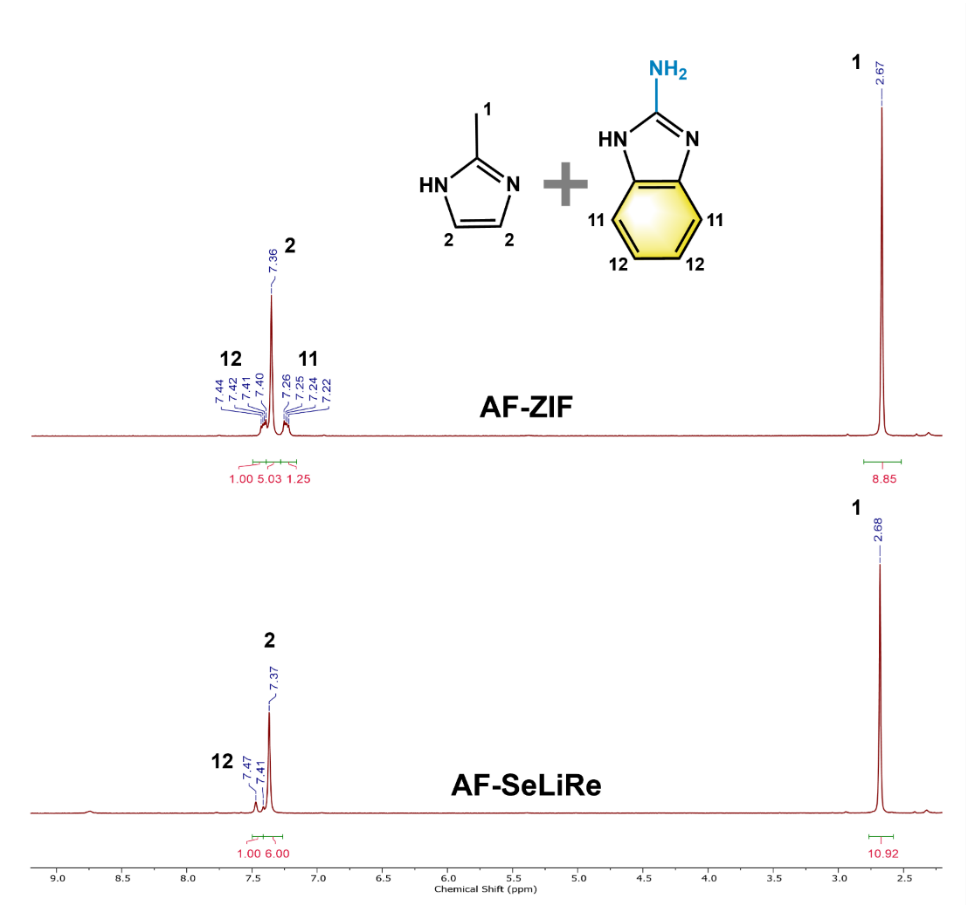


**Figure S7.** $^{1}$H NMR of AF-ZIF and AF-SeLiRe.

AF-ZIF: *$^{1}$H NMR (250 MHz, Acetic Acid-$d_4$) δ 7.76 (d, J = 4.0 Hz, 1H), 7.49 (dd, J = 6.1, 3.2 Hz, 1H), 7.37 (s, 4H), 2.89 (s, 2H), 2.68 (s, 7H).* AF-SeLiRe: *$^{1}$H NMR (250 MHz, Acetic Acid-$d_4$) δ 7.75 (s, 1H), 7.47 (dd, J = 6.1, 3.2 Hz, 1H), 7.35 (s, 4H), 2.87 (s, 2H), 2.66 (s, 8H).*

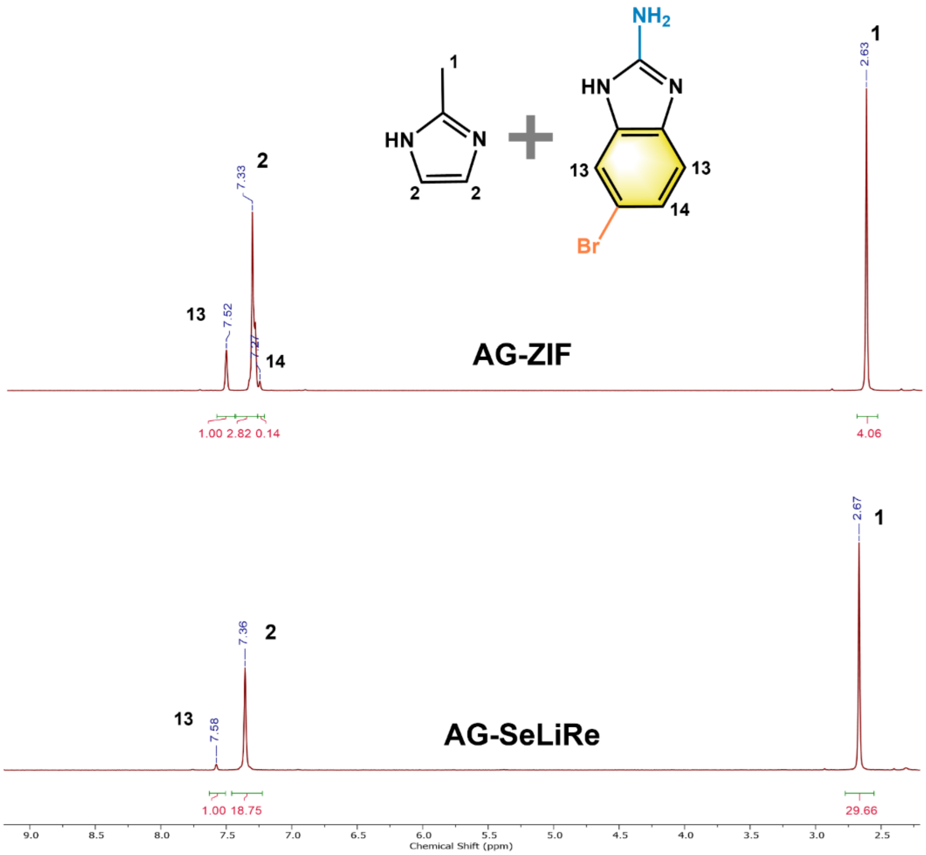


**Figure S8.** $^1$H NMR of AG-ZIF and AG-SeLiRe.

AG-ZIF: *$^1$H NMR (250 MHz, Acetic Acid-$d_4$) δ 7.52 (s, 1H), 7.33 (s, 3H), 2.63 (s, 4H).* AG-SeLiRe: *$^1$H NMR (250 MHz, Acetic Acid-$d_4$) δ 7.58 (s, 1H), 7.36 (s, 19H), 2.67 (s, 30H).*

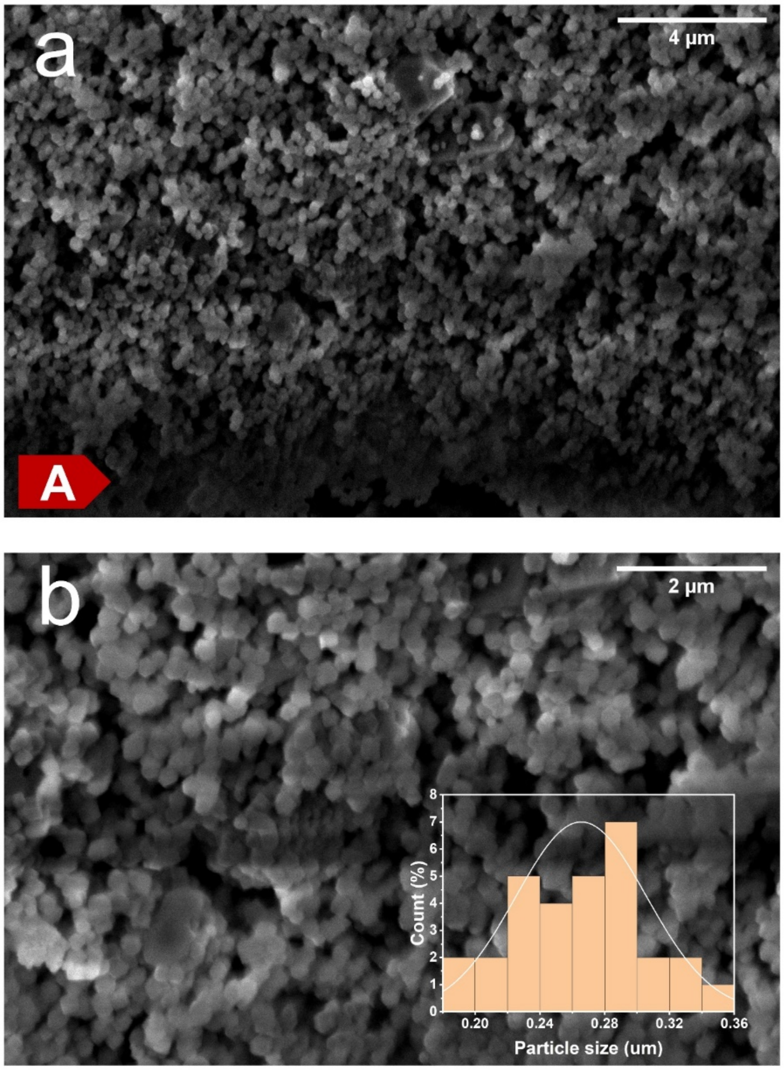


**Figure S9.** (**a**) SEM image of A-ZIF. (**b**) Enlarged SEM image of A-ZIF. An ImageJ program was used to select 30 particles from each sample.

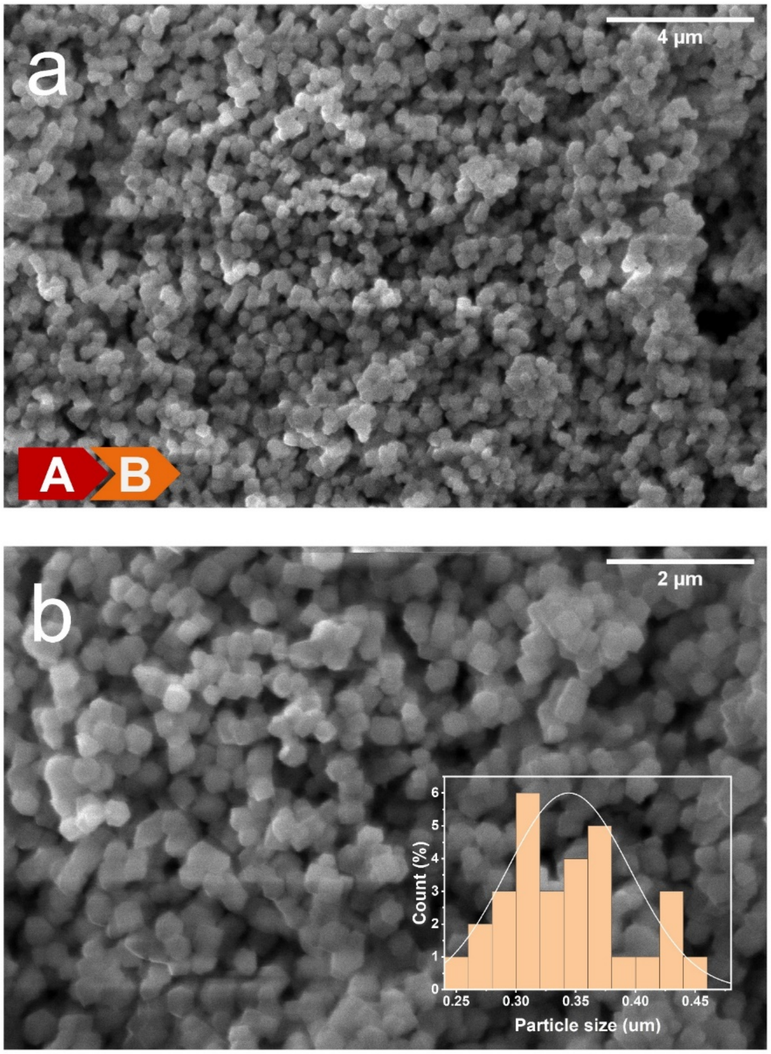


**Figure S10.** (**a**) SEM images of AB-ZIF. (**b**) Enlarged SEM images of AB-ZIF. An ImageJ program was used to select 30 particles from each sample.

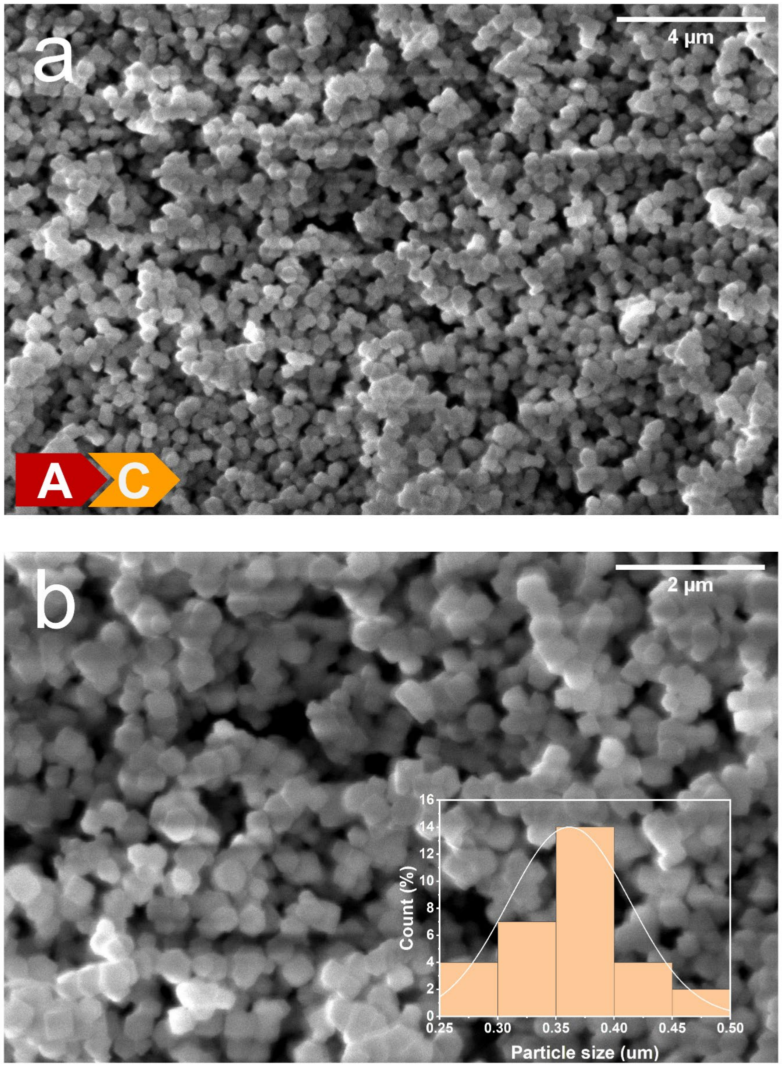


**Figure S11.** (**a**) SEM image of AC-ZIF. (**b**) Enlarged SEM image of AC-ZIF. An ImageJ program was used to select 30 particles from each sample.

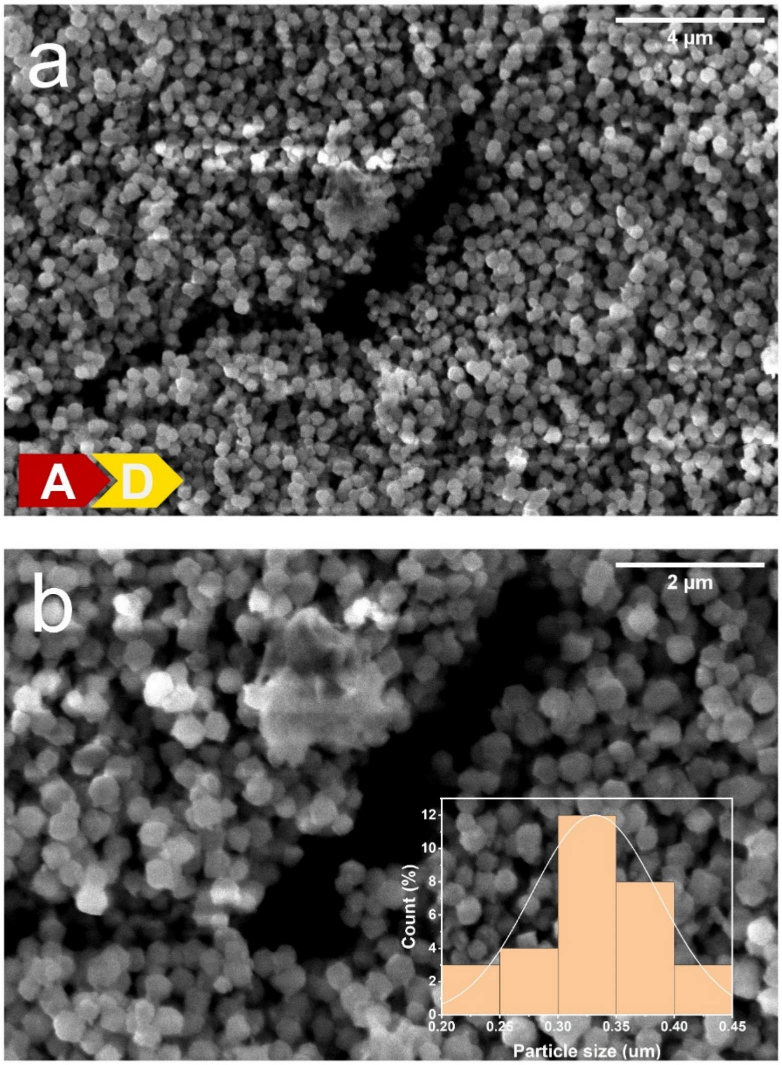


**Figure S12.** (**a**) SEM image of AD-ZIF. (**b**) Enlarged SEM image of AD-ZIF. An ImageJ program was used to select 30 particles from each sample.

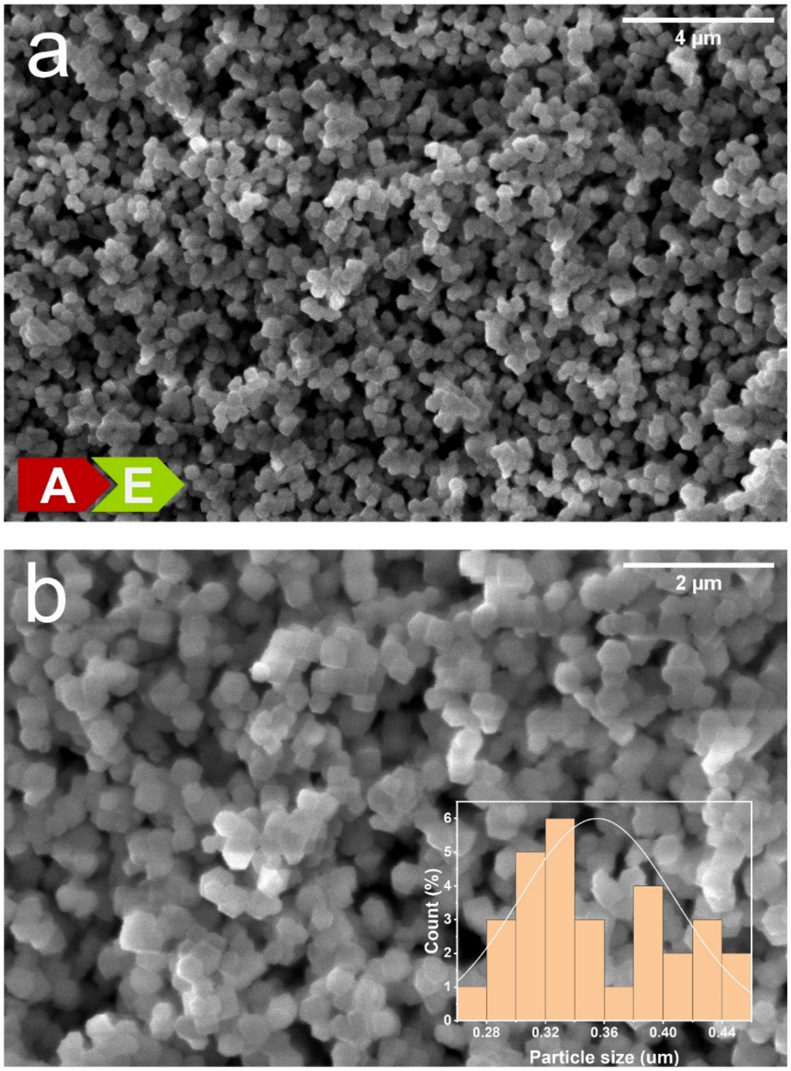


**Figure S13.** (**a**) SEM image of AE-ZIF. (**b**) Enlarged SEM image of AE-ZIF. An ImageJ program was used to select 30 particles from each sample.

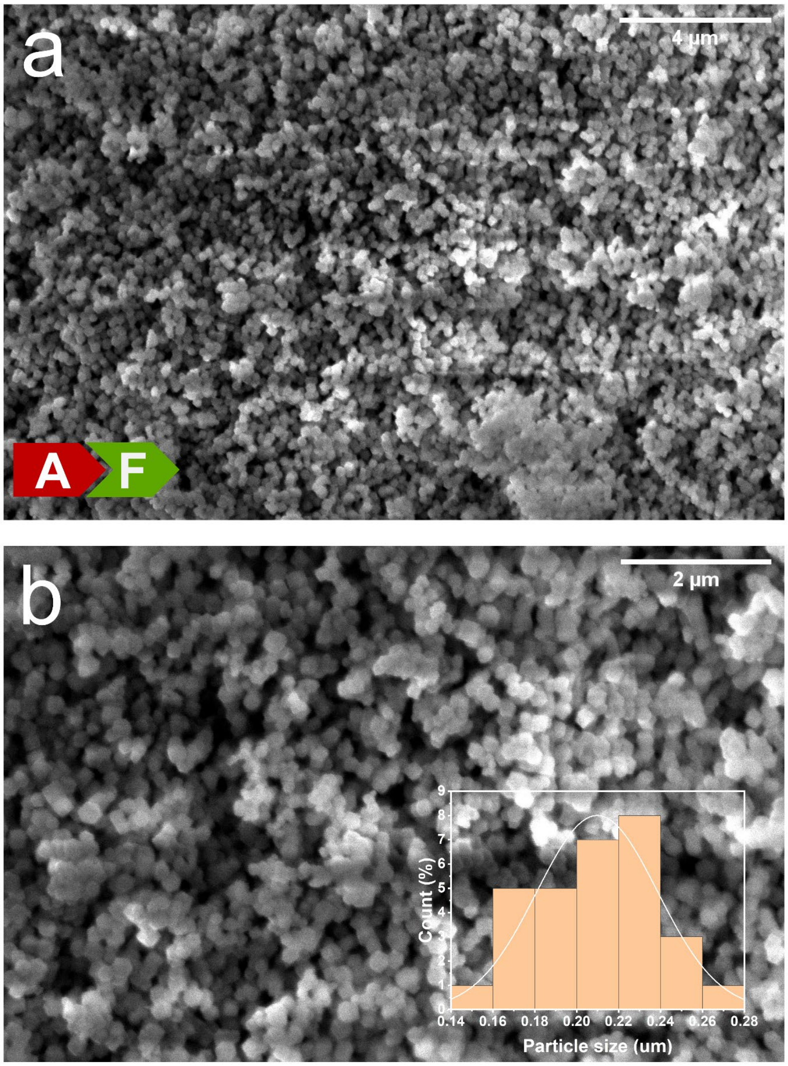


**Figure S14.** (**a**) SEM image of AF-ZIF. **b**, Enlarged SEM image of AF-ZIF. An ImageJ program was used to select 30 particles from each sample.

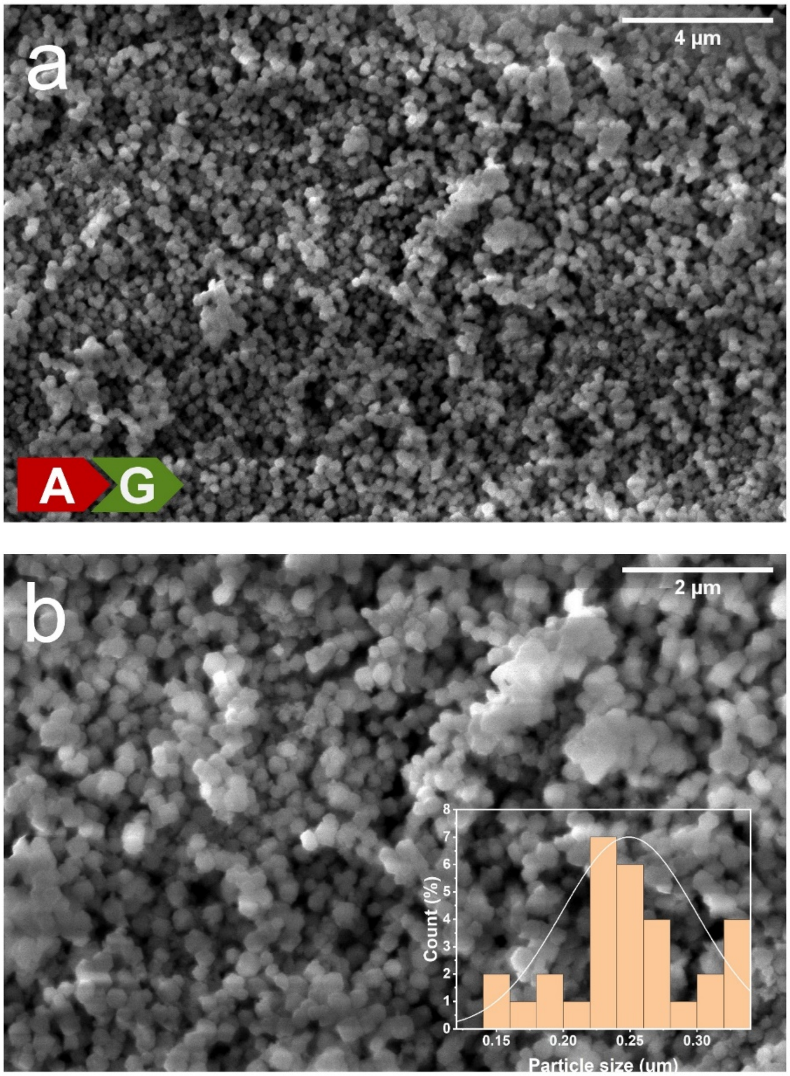


**Figure S15.** (**a**) SEM image of AG-ZIF. (**b**) Enlarged SEM image of AG-ZIF. An ImageJ program was used to select 30 particles from each sample.

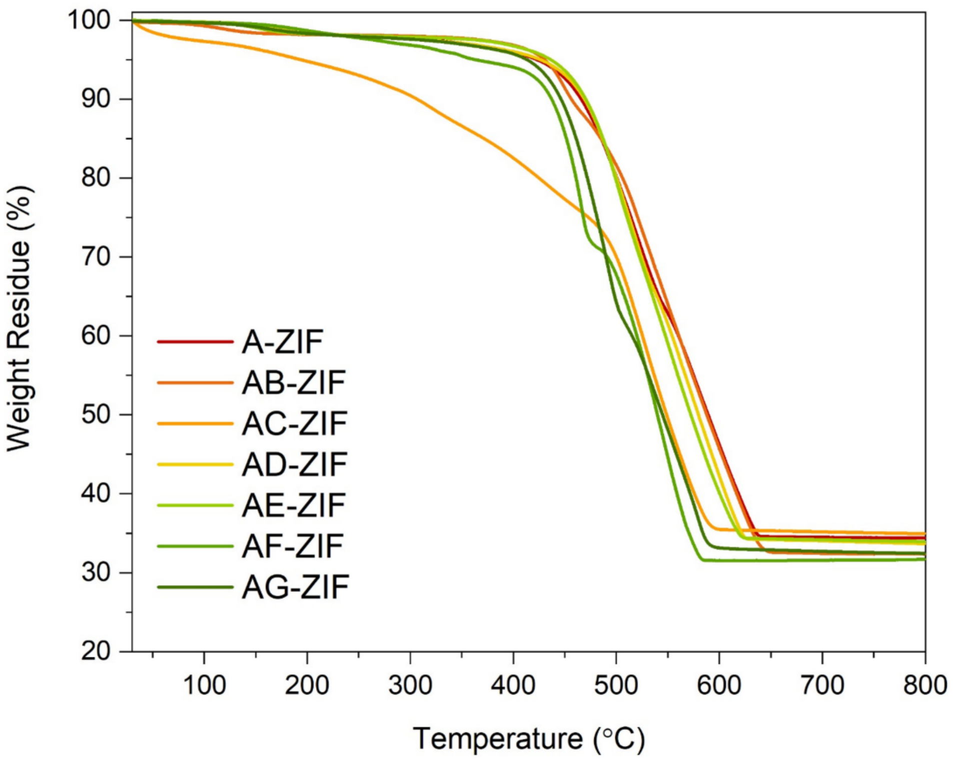


**Figure S16.** TGA curves of A-ZIF, AB-ZIF, AC-ZIF, AD-ZIF, AE-ZIF, AF-ZIF and AG-ZIF. All TGA tests were carried out in an argon atmosphere.

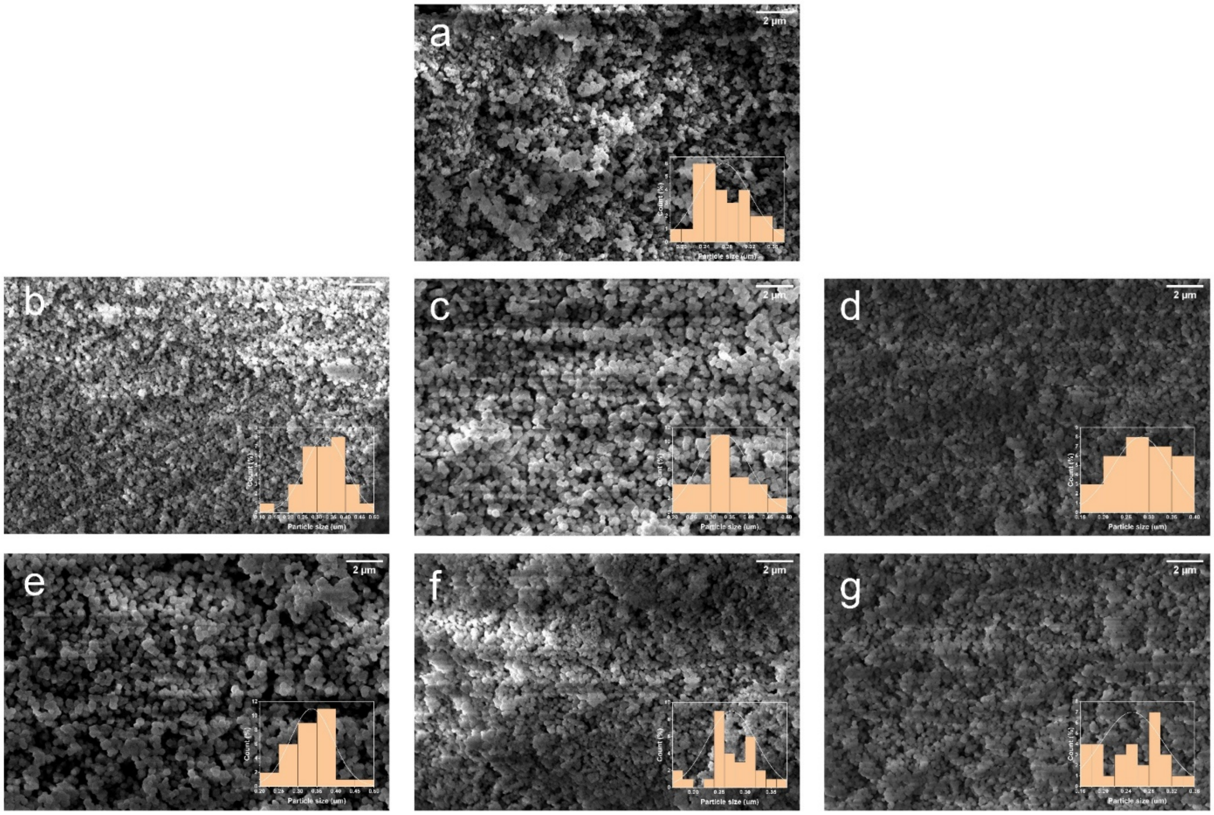


**Figure S17.** SEM images of A-Heating (**a**), AB-SeLiRe (**b**), AC-SeLiRe (**c**), AD-SeLiRe (**d**), AE-SeLiRe (**e**), AF-SeLiRe (**f**) and AG-SeLiRe (**g**). An ImageJ program was used to select 30 particles from each sample.

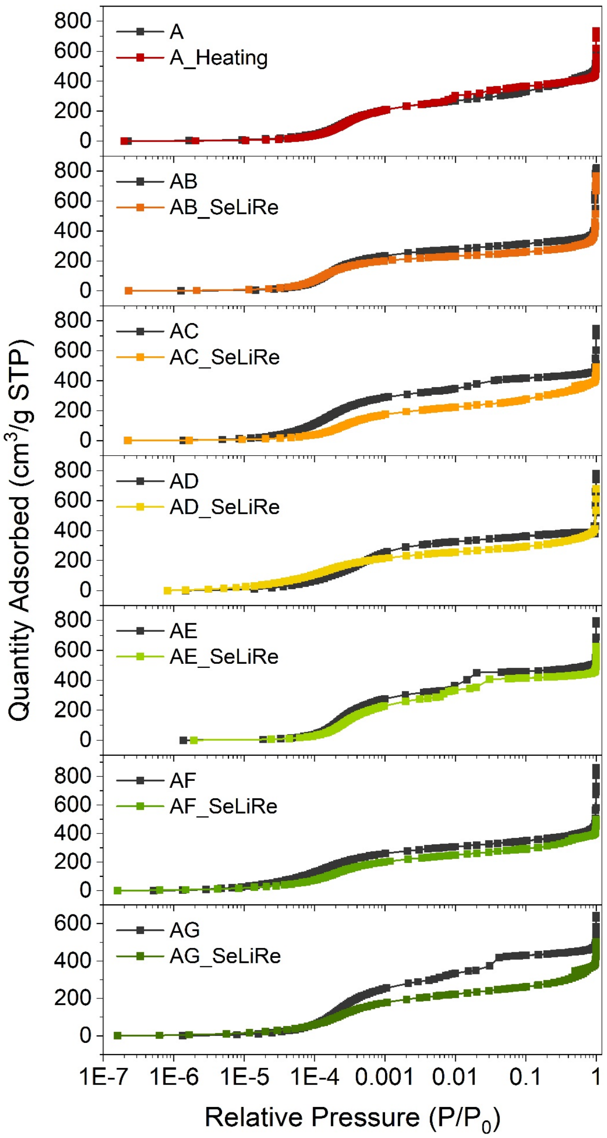


**Figure S18.** Semi-log plot of $N_2$ physisorption isotherms of A-ZIF, A-Heating, AB-ZIF, AB-SeLiRe, AC-ZIF, AC-SeLiRe, AD-ZIF, AD-SeLiRe, AE-ZIF, AE-SeLiRe, AF-ZIF, AF-SeLiRe, AG-ZIF, and AG-SeLiRe.

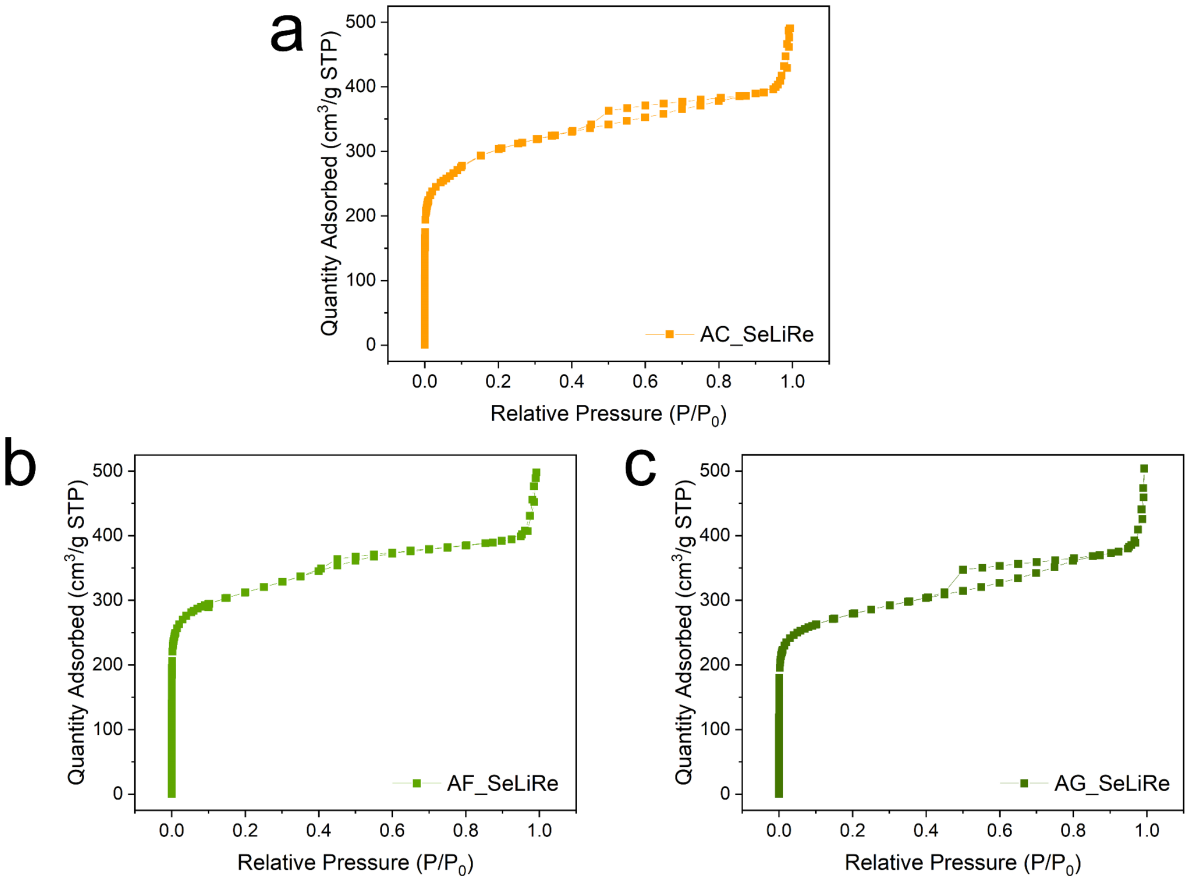


**Figure S19.** $N_2$ physisorption isotherms with the hysteresis loop of AC-SeLiRe (**a**), AF-SeLiRe (**b**) and AG-SeLiRe (**c**).

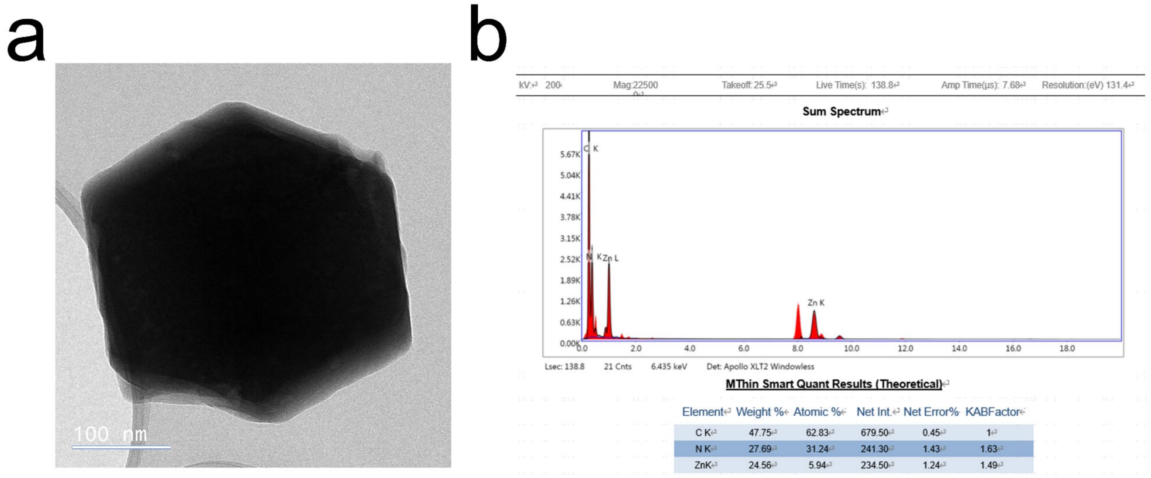


**Figure S20.** (**a**) TEM image of single-particle AG-ZIF. (**b**) EDS quantitative elemental analysis of single-particle AG-SeLiRe.

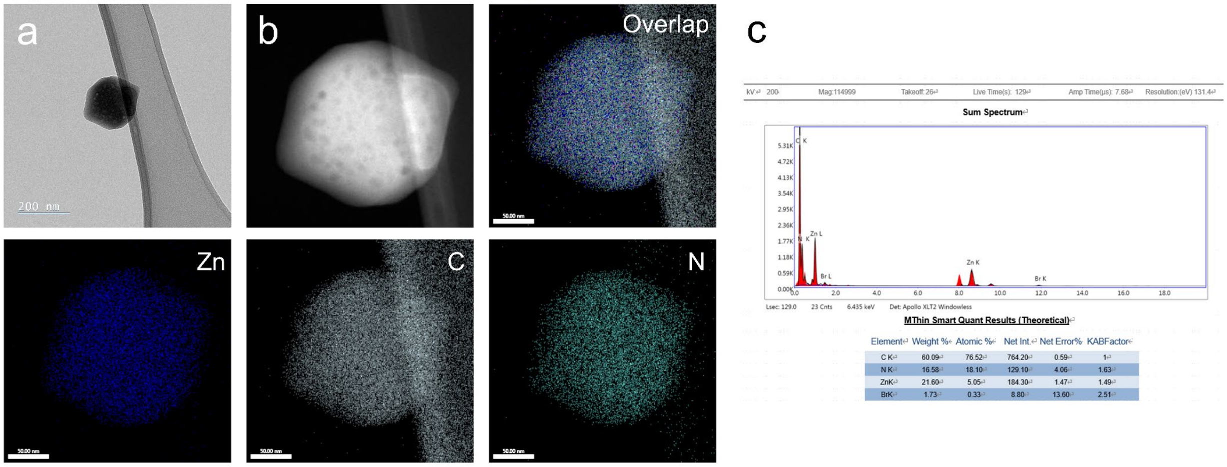


**Figure S21.** (**a**) TEM image of single-particle AC-SeLiRe. (**b**) Elemental mapping of single-particle AC-SeLiRe. (**c**) EDS quantitative elemental analysis of single-particle AC-SeLiRe.

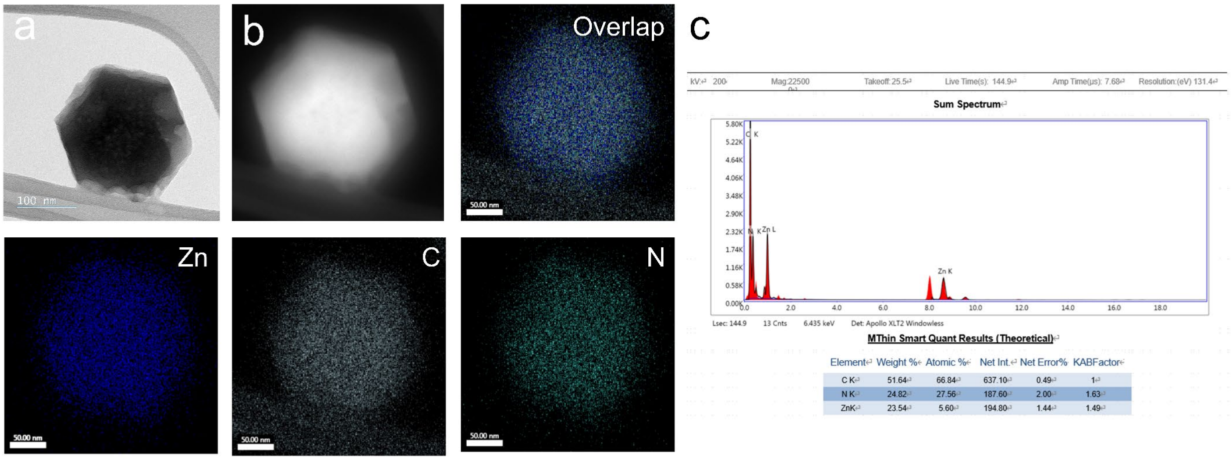


**Figure S22.** (**a**) TEM image of single-particle AF-SeLiRe. (**b**) Elemental mapping of single-particle AF-SeLiRe. (**c**) EDS quantitative elemental analysis of single-particle AF-SeLiRe.

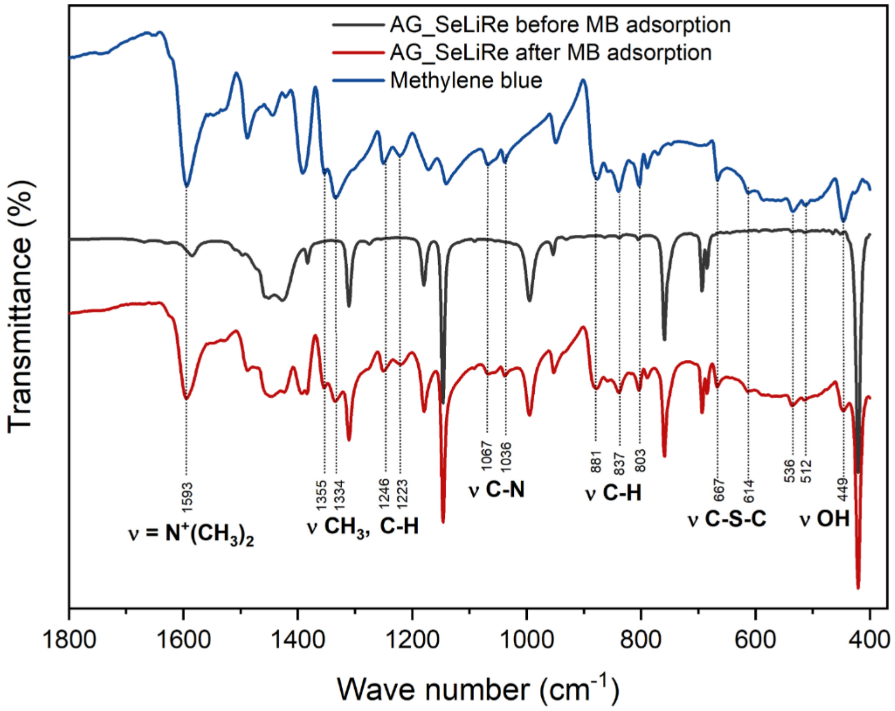


**Figure S23.** ATR-IR spectra of AG-SeLiRe before and after saturation of methylene blue adsorption.

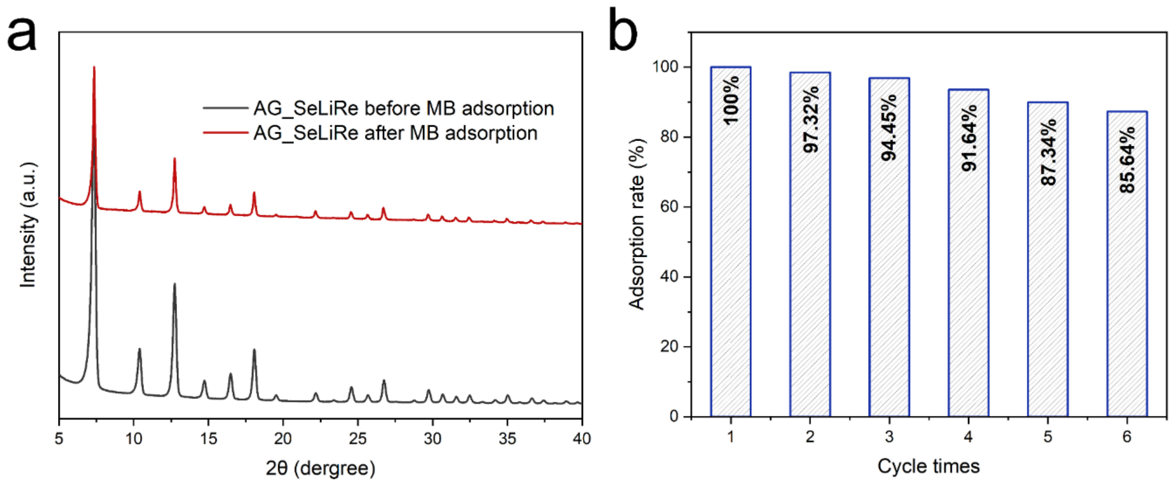


**Figure S24.** (**a**) XRD patterns of AG-SeLiRe before and after saturation of methylene blue adsorption. (**b**) Adsorption-desorption cycles with AG-SeLiRe, the methylene blue adsorption reached saturation in each cycle.

**Table S1.** Calculation of secondary ligands B-F ratio in ML-ZIFs by $^{1}$H NMR.

| Samples | AB-ZIF | AC-ZIF | AD-ZIF | AE-ZIF | AF-ZIF | AG-ZIF |
|---|---|---|---|---|---|---|
| **Synthetic mass of secondary ligand/ primary ligand (g/g)** | 0.6/1.4 | | | | | |
| **Synthetic ratio of secondary ligand (mol%)** | 19.3% | 29.5% | 22.1% | 21.0% | 21.0% | 14.2% |
| **Actual ratio of secondary ligand (mol%)** | 19.5% | 13.1% | 27.3% | 26.0% | 13.9% | 14.7% |
| **Actual ratio of secondary ligand after SeLiRe (mol%)** | 19.0% | ~ 0% | 27.2% | 24.6% | 5.5% | 2.0% |

**Table S2.** Specific surface area and porosity parameters of ML-ZIFs and SeLiRe-ZIFs.

| **Samples** | **Specific surface area[a] ($m^2\,g^{-1}$)** | **Total Pore Volume ($cm^3\,g^{-1}$)** | **Micropore Volume[b] ($cm^3\,g^{-1}$)** | **Mesopore Volume[c] ($cm^3\,g^{-1}$)** |
|---|---|---|---|---|
| A-ZIF | 1315.0 | 0.518 | 0.518 | nonporous |
| A-Heating | 1169.2 | 0.494 | 0.436 | 0.056 |
| AB-ZIF | 1255.4 | 0.429 | 0.429 | nonporous |
| AB-SeLiRe | 1041.9 | 0.357 | 0.357 | nonporous |
| AC-ZIF | 1309.9 | 0.629 | 0.565 | 0.064 |
| AC-SeLiRe | 1104.2 | 0.504 | 0.273 | 0.231 |
| AD-ZIF | 1022.3 | 0.435 | 0.435 | nonporous |
| AD-SeLiRe | 807.7 | 0.504 | 0.411 | 0.093 |
| AE-ZIF | 1291.3 | 0.544 | 0.544 | nonporous |
| AE-SeLiRe | 1087.6 | 0.457 | 0.375 | 0.082 |
| AF-ZIF | 1192.9 | 0.499 | 0.499 | nonporous |
| AF-SeLiRe | 1180.3 | 0.540 | 0.342 | 0.198 |
| AG-ZIF | 1381.2 | 0.660 | 0.634 | 0.026 |
| AG-SeLiRe | 1053.6 | 0.522 | 0.334 | 0.188 |

[a] Brunauer-Emmett-Teller specific surface area.

[b] Cumulative adsorption volume of micropores from 0 to 2 nm in diameter.

[c] Cumulative adsorption volume of mesopores from 2 to 50 nm in diameter according to International Union of Pure and Applied Chemistry (IUPAC).

**Table S3.** Kinetics parameters of ML-ZIFs and SeLiRe-ZIFs.

| Adsorbents | $Q_{e.exp}$ /mg $g^{-1}$ | Pseudo-first-order model | | | Pseudo-second-order model | | |
|---|---|---|---|---|---|---|---|
| | | $Q_{e.cal}$ /mg $g^{-1}$ | $K_1$ /$h^{-1}$ | $R^2$ | $Q_{e.cal}$ /mg $g^{-1}$ | $K_2$ / g $mg^{-1}$ $h^{-1}$ | $R^2$ |
| A-ZIF | 0.514 | 0.499 | 3.83 | 0.9949 | 0.552 | 9.13 | 0.9843 |
| A-Heating | 0.943 | 0.937 | 6.63 | 0.9993 | 1.00 | 10.1 | 0.9877 |
| AB-ZIF | 2.76 | 2.71 | 7.39 | 0.9962 | 2.89 | 3.95 | 0.9912 |
| AB-SeLiRe | 2.54 | 2.53 | 7.04 | 0.9962 | 2.70 | 3.96 | 0.9845 |
| AC-ZIF | 4.42 | 4.21 | 7.73 | 0.9745 | 4.51 | 2.58 | 0.9968 |
| AC-SeLiRe | 24.4 | 23.5 | 6.23 | 0.9953 | 25.2 | 0.373 | 0.9936 |
| AD-ZIF | 3.01 | 2.93 | 5.06 | 0.9869 | 3.18 | 2.30 | 0.9961 |
| AD-SeLiRe | 6.56 | 6.34 | 7.60 | 0.9962 | 6.74 | 1.78 | 0.9937 |
| AE-ZIF | 5.98 | 5.70 | 5.95 | 0.9800 | 6.13 | 1.45 | 0.9974 |
| AE-SeLiRe | 4.88 | 4.72 | 8.23 | 0.9886 | 5.00 | 2.67 | 0.9981 |
| AF-ZIF | 3.85 | 3.73 | 6.97 | 0.9939 | 3.99 | 2.66 | 0.9937 |
| AF-SeLiRe | 25.6 | 24.9 | 5.83 | 0.9978 | 26.9 | 0.316 | 0.9817 |
| AG-ZIF | 4.14 | 4.52 | 6.04 | 0.9842 | 4.90 | 1.79 | 0.9986 |
| AG-SeLiRe | 30.5 | 29.2 | 5.69 | 0.9953 | 31.6 | 0.261 | 0.9921 |

**Table S4.** Isotherm parameters of ML-ZIFs and SeLiRe-ZIFs.

| **Adsorbents** | **Langmuir model** | | | **Freundlich model** | | |
|---|---|---|---|---|---|---|
| | $Q_{m.cal}$ /mg $g^{-1}$ | $K_L$ /L $mg^{-1}$ | $R^2$ | n | $K_F$ / (mg $g^{-1}$) (mg $L^{-1}$) $^{-n}$ | $R^2$ |
| A-ZIF | 0.531 | 0.966 | 0.9801 | 3.04 | 0.244 | 0.9158 |
| A-Heating | 0.969 | 1.45 | 0.9960 | 3.55 | 0.523 | 0.9066 |
| AB-ZIF | 3.01 | 0.762 | 0.9986 | 2.69 | 1.23 | 0.9264 |
| AB-SeLiRe | 2.70 | 0.909 | 0.9886 | 2.89 | 1.19 | 0.9221 |
| AC-ZIF | 4.46 | 0.734 | 0.9966 | 2.51 | 1.76 | 0.9448 |
| AC-SeLiRe | 22.4 | 2.88 | 0.9954 | 14.9 | 2.80 | 0.9407 |
| AD-ZIF | 2.78 | 1.75 | 0.9973 | 3.78 | 1.56 | 0.8875 |
| AD-SeLiRe | 6.95 | 1.19 | 0.9944 | 2.75 | 3.37 | 0.9468 |
| AE-ZIF | 6.35 | 0.768 | 0.9978 | 2.36 | 2.52 | 0.9523 |
| AE-SeLiRe | 5.16 | 1.08 | 0.9947 | 2.80 | 2.42 | 0.9380 |
| AF-ZIF | 3.87 | 1.45 | 0.9960 | 3.55 | 2.09 | 0.9066 |
| AF-SeLiRe | 26.4 | 3.48 | 0.9969 | 18.4 | 2.83 | 0.9411 |
| AG-ZIF | 4.67 | 0.993 | 0.9814 | 2.77 | 2.12 | 0.9445 |
| AG-SeLiRe | 28.1 | 3.52 | 0.9958 | 20.4 | 2.63 | 0.9466 |

**X-ray Diffraction (XRD).** The analysis was conducted using a PANalytical X'Pert Pro multi-purpose diffractometer (MPD) in Bragg-Brentano geometry, featuring a Cu anode operated at 45 kV and 40 mA. The setup included a BBHD Mirror and an X-Celerator multichannel detector. Diffraction patterns were recorded within a 2θ range of 5° to 90°. All data were collected using Cu $K_\alpha$ and $K_\beta$ radiation (2:1 ratio) at a wavelength of 1.54060 Å and a scanning speed of 0.5° $min^{-1}$. The sample was affixed to a single-crystal silicon holder with a drop of heptane and rotated every 4 seconds during measurement.

**Attenuated total reflection-infrared spectroscopy (ATR-IR).** Spectra obtained with Perkin Elmer Spectrum two FT-IR spectrometers were in the infrared range of 400-4000 $cm^{-1}$, with the $LiTaO_3$ (lithium tantalate) MIR detector. Control each data Force Gauge at around 120 with accumulation number of scans is 8 times.

**Nitrogen Physisorption.** Measurements were carried out at 77 K on a Micromeritics 3Flex analyzer. Prior to analysis, samples underwent vacuum outgassing at 150°C for 3-12 hours. Surface area determinations were based on the Brunauer-Emmet-Teller (BET) method, following best practices for microporous materials. Pore size distribution data were derived from the adsorption isotherm branch, using nonlocal density functional theory (NLDFT) modeling.

**Scanning electron microscope (SEM).** Imaging was performed on a FEI Quanta 250 (Schottky-)FEG-SEM with the support of USTEM at TU Wien. The device, equipped with an ETD secondary electron detector and an EDAX-AMETEK Octane Elite 55 detector, facilitated elemental characterization via energy-dispersive X-ray spectroscopy (EDS). Operated at 10 kV and a 5 mm working distance, the instrument achieved a maximum resolution of ~2 nm. X-ray microanalysis was performed at 20 kV and a working distance of 10 mm.

**Transmission electron microscope (TEM).** High-resolution TEM (HR-TEM) analyses presented in this work were conducted using a Tecnai F20 FEG-TEM, also facilitated by USTEM at TU Wien. This

microscope was equipped with an X-FEG source, Gatan Rio16 CCD-camera, DigiSTEM II with HAADF detector for STEM imaging, and an EDAX-AMETEK Apollo XLTW SDD EDX detector. The instrument was operated at 200 kV throughout, achieving a lattice resolution limit of ~0.14 nm.

**Nuclear magnetic resonance spectroscopy ($^{1}$H NMR).** Liquid phase $^{1}$H spectra were measured using the Bruker ADVANCE 250 (250.13 MHz) instrument, which is equipped with a 5 mm inverse-broad probe head and z-gradient unit. Due to the limited solubility of ZIFs in typical NMR solvents, the sample weighing 2 mg was digested with 0.5 ml of $d_4$-acetic acid and then sonicated until it was well dispersed in the acid.

**Thermogravimetric analysis (TGA).** Measurements were carried out using PerkinElmer 8000 (Waltham, USA) with the sample heated in of $Al_2O_3$ crucible. The heating rate was 10°C/min, either in air, argon and nitrogen flow, and finally held at 600°C for 1 hour.
